# Clustering of Subgingival Microbiota Reveals Microbial Disease Ecotypes Associated with Clinical Stages of Periodontitis in a Cross-Sectional Study

**DOI:** 10.3389/fmicb.2017.00340

**Published:** 2017-03-01

**Authors:** Sébastien Boutin, Daniel Hagenfeld, Heiko Zimmermann, Nihad El Sayed, Tanja Höpker, Halina K. Greiser, Heiko Becher, Ti-Sun Kim, Alexander H. Dalpke

**Affiliations:** ^1^Department of Infectious Diseases, Medical Microbiology and Hygiene, University Hospital HeidelbergHeidelberg, Germany; ^2^Translational Lung Research Center Heidelberg (TLRC), German Center for Lung Research (DZL)Heidelberg, Germany; ^3^Department of Periodontology and Restorative Dentistry, University Hospital MünsterMünster, Germany; ^4^Section of Periodontology, Department of Conservative Dentistry, University Hospital HeidelbergHeidelberg, Germany; ^5^Institute of Public Health, Epidemiology and Biostatistics, Heidelberg UniversityHeidelberg, Germany; ^6^Division of Cancer Epidemiology, German Cancer Research CenterHeidelberg, Germany; ^7^Institute of Medical Biostatistics and Epidemiology, University Hospital Hamburg-EppendorfHamburg, Germany

**Keywords:** periodontitis, microbiome, subgingival plaque, microbial ecology, polymicrobial infections

## Abstract

Periodontitis is characterized by chronic inflammation associated with alteration of the oral microbiota. In contrast to previous microbiome studies focusing *a priori* on comparison between extreme phenotypes, our study analyzed a random sample of 85 people. The aim of this study was to link microbial differences to disease’s prevalence and severity. Using next generation sequencing of 16S rRNA amplicons and cluster analysis, we observed that the population can be divided into two major ecotypes: One mainly contained periodontal healthy/mild periodontitis individuals whereas the second ecotype showed a heterogeneous microbial distribution and clustered into three distinct sub-ecotypes. Those sub-ecotypes differed with respect to the frequency of diseased patients and displayed a gradual change in distinct subgingival microbiota that goes along with clinical disease symptoms. In ecotype 2, the subgroup with no clinical signs of disease was linked to an increase of *F. nucleatum vincentii* but also several other species, while only in “end-stage” dysbiosis classical red complex bacteria gained overweight. Therefore, the microbial disease ecotypes observed in our population can lead to an establishment of an early microbial risk profile for clinically healthy patients.

## Introduction

Periodontitis is a chronic inflammatory disease caused by a dysbiosis of the oral microbiota (Darveau, 2010; Yost et al., 2015) and by the host response to these alterations (Costalonga and Herzberg, 2014). In the United States, 46% of adults aged ≥ 30 years suffer from periodontitis and approximately 10% of the population have deep periodontal pockets, worldwide (Page and Eke, 2007; Petersen and Ogawa, 2012; Eke et al., 2015). The oral cavity and especially the periodontal pocket provides a unique eco-system for microbial organisms and harbors a diverse microbiota with up to 700 prokaryote species (Chen et al., 2010). One model for pathogenesis of periodontitis suggests that periodontal microbial communities can be clustered into complexes that are associated with disease severity (Socransky et al., 1998). Seminal work, at that time based on culture dependent techniques, of Socransky identified a “red complex” harboring *Porphyromonas gingivalis, Tannerella forsythia*, and *Treponema denticola*, associated with the severe form of periodontitis. Further complexes included an intermediate orange complex with, e.g., *Fusobacterium nucleatum* and a yellow and green complex dominated by *Streptococcus* species, the latter being associated with health. Support for a classical role for the red complex as direct pathogens came from the observation by Holt showing induction of periodontitis upon oral implantation of these bacteria in non-human primates (Holt et al., 1988).

However, more recent concepts suggest that keystone pathogens can disrupt tissue homeostasis and change the composition of the commensal microbiota thereby generating host immune modulation and dysbiosis, that is responsible for periodontitis (Hajishengallis et al., 2011, 2012). Such a concept takes into account observations that periodontal pathogens often are low abundant and can be present in healthy people (Haffajee et al., 1998). Periodontitis resembles the process of microbial succession with an increase of periodontitis-associated taxa while health-associated species remain but decrease in number. In turn, the microbial community structure changes significantly, and biomass typically increases.

With the establishment of NGS, a nearly unbiased view of the bacterial composition is accessible. Conventional techniques are limited to cultivable organisms or a pre-selection of targeted pathogens is needed (Socransky et al., 1998, 2013). Using NGS, usually a part of the 16S rRNA gene is amplified and sequenced for taxonomic characterization of a bacterial community. Due to progress in sequencing efficiency and costs it is now possible to use NGS in population based studies to monitor larger cohorts.

Using NGS, comparisons between individuals with and without periodontitis revealed different microbiological compositions in periodontitis and health. *Fusobacteria*, *Porphyromonas*, *Tannerella*, and *Treponema* were increased in periodontitis. *Streptococcus*, *Firmicutes*, and *Proteobacteria* were associated with periodontal health (Griffen et al., 2012; Wichmann et al., 2012; Abusleme et al., 2013; Li et al., 2014). Communities in health and periodontitis differed, with higher diversity and biomass in periodontitis (Wichmann et al., 2012; Abusleme et al., 2013; Hong et al., 2015). Subgingival clusters in periodontally diseased patients were not associated with demographic, medical or disease-specific clinical parameters other than periodontitis extent (Li et al., 2014). However, most of the actual studies focus on extremes of the disease, mostly healthy versus severe disease. Cluster analysis with such a pre-selection could result in a bias, neglecting intermediate states of disease. Furthermore, population based studies with higher sample sizes are missing so far.

The objective of this study was primarily to identify the variation in the microbial complexes of subgingival samples from a population-based study and secondly to identify the relationship of oral health variables with the microbial composition within these complexes.

## Materials and Methods

### Description of the Cohort

The NAKO aims at recruiting a representative sample from the general population in Germany (Wichmann et al., 2012). Recruitment takes place in 18 study centers distributed throughout Germany and will include 200,000 people aged between 20 and 69 years. Feasibility studies were conducted in all centers in 2012 to test specific aspects of the NAKO (Zimmermann et al., 2015). The aim of one feasibility study was a comparison of oral health and systemic parameters of Turks, Germans and Resettlers from the former Soviet Union (Resettlers) in the Rhine-Neckar metropolitan region. Since for this study both Germans and persons with a migration background were selected, different recruitment channels were chosen (Reiss et al., 2014). Briefly, on the one hand, people were drawn randomly based on the nationality from registration offices and on the other hand network recruitment strategies were used. For microbial analysis, participants (*n* = 85) from the study center Heidelberg were selected.

### Periodontal Examination

Two calibrated dentists DH, NE (with minimal concordance of 90% in periodontal probing within an error interval of 1 mm) performed full-mouth periodontal examination at six sites per tooth, except third molars. AL was measured from the cemento-enamel junction or from the margin of the restoration to the bottom of the pocket. PD was measured from the gingival margin to the bottom of the pocket. BOP was measured as presence or absence of bleeding after probing (Lang et al., 1986). The periodontal examination was conducted using a UNC-PCP15 Color-Coded Probe (Hu-Friedy Europe, Rotterdam/Netherlands) with a black band for each millimeter up to 15 mm. Subjects with periodontitis were defined according to the CDC-AAP classification (Page and Eke, 2007). Briefly, participants with two or more approximal sites with AL of 4 mm or higher, but not on the same tooth or two or more sites with PD 5 mm or higher, but not on same tooth were classified as having moderate to severe periodontitis. All other participants were classified as mild or no periodontitis. One subgingival plaque sample per patient was taken from the mesio-buccal site of the first molar of a randomized quadrant. Randomization was performed with a randomization list. If the first molar was missing, the next mesial tooth in the same jaw and side was selected for plaque sampling. As a consequence of the randomized selection of the sampling site, the chosen site did not necessarily reflect the disease state of the host. In seven cases subjects had a full mouth diagnosis of moderate/severe periodontitis, but sampling occurred on teeth with no AL and presenting physiologic PD (defined as AL < 4 mm and PD < 4mm). At these sites it is more likely to find an unchanged microbiome (Griffen et al., 2012; Shi et al., 2015). Thus, for these cases the samples were classified as having no or mild periodontitis.

### Plaque Sampling

Subgingival plaque was sampled from the mesio-buccal site of the first molar of a randomized quadrant. Randomization was performed with a randomization list. If the molar was missing, the next mesial tooth in the same jaw and side was selected for plaque sampling. Firstly, visible supra-gingival plaque was removed from the sampling tooth with a sterile cotton pellet and secondly, aROEKO ISO25 sterile paper point (Coltene, Altstätten, Switzerland) was introduced to the bottom of pocket and remained there for 10 s. Plaque samples were placed in 1.5 ml safe-lock Eppendorf tubes and placed immediately at -20°C until DNA extraction.

### DNA Extraction

DNA extractions were performed using the QIAamp Mini Kit (QIAGEN, Hilden, Germany). Protease solution (7.2 mAU) and 200 μL of Buffer AL were added to the sample followed by a 15 s vortex. Samples were incubated at 56°C for 10 min and then purified according to the manufacturer’s protocol. DNA was eluted by adding 100 μL of buffer AE to the column, incubation for 1 min at room temperature and centrifugation at 6000 × *g* for 1 min. Negative controls were performed by doing the extraction without clinical samples to control for absence of contamination.

### Quantitative PCR

Quantitative PCRs were processed to evaluate the number of 16S rRNA gene copies using Unibac primer (forward: 5′-TGG AGC ATG TGG TTT AAT TCG A-3′; reverse: 5′-TGC GGG ACT TAA CCC AAC A-3′) targeting the 16S rRNA gene. PCR reactions were performed in a final volume of 15 μL composed of 1X Sybr-green mastermix (Life technology, Darmstadt, Germany), 50 pmol of each primer and 2 μL of DNA (or plasmid DNA standards). The thermal cycler conditions were: a first denaturation at 95°C for 20 s, 40 amplification cycles (95°C for 3 s, 60°C for 30 s) and two final steps at 95°C for 15 s and 60°C for 1 min followed by a melt curve. All reactions were performed in duplicate in a StepOnePlus Real-time PCR system (Applied Biosystems, Foster City, CA, USA). Quantification of the 16S rRNA gene’s number of copies was performed by comparison to the Cycle threshold value of a plasmid DNA standard, which had been quantified by spectrophotometry.

### Library Preparation for Next Generation Sequencing (NGS)

DNA was amplified using universal bacterial primers flanking the V4 region [515F and 806R from (Caporaso et al., 2012)]. Each primer was tagged with an individual barcode to assign the sequences to the samples. PCR reactions were performed in 25 μL volumes composed of Q5 High-Fidelity 1X Master Mix (New England Biolabs GmbH, Germany), 0.5 μM of each primer and 2 μL of DNA. The thermal cycler (Primus 25, Peqlab Biotechnologie GmbH, Germany or FlexCycler^2^, Analytik Jena AG, Germany) conditions were: a first denaturation at 94°C for 3 min, 30 amplification cycles (94°C for 45 s, 50°C for 1 min, and 72°C for 1 min 30 s) and a final extension at 72°C for 10 min. Negative controls were performed using the negative control from the extraction step and using sterile water as template. PCR products were then checked by agarose gel electrophoresis (2%) for presence of amplicons. Both negative controls were negative on the gel. Amplicons were then purified by using Agencourt AMPure XP beads (Beckman Coulter, Germany) following the manufacturer’s instructions. Purified products were checked for quality and concentration using a ND-1000 Nanodrop instrument (Nanodrop, Wilmington, DE, USA) and Bioanalyzer (Agilent Technologies Inc., Böblingen, Germany). Equimolar mix of all the PCR products was then sent to the Center for “Quantitative Analysis of Molecular and Cellular Biosystems” at Heidelberg University (BIOquant) which performed the ligation of the sequencing adapters to the library and the paired-end sequencing on an Illumina Miseq sequencing system with 250 cycles.

### Analysis of Sequences

Paired sequences were assembled in contigs with stringent parameters: minimum overlap of 100 nt and maximum mismatch of 5 nt. Contigs were then filtered for quality; sequences with a quality score lower than 30 over 97% of the length were discarded. Each contig was assigned to the sample with the barcodes on the right and left ends (allowing no mismatch per barcode). The assignment and the pre-treatment of the sequences were done using MOTHUR software (Schloss et al., 2009). Sequences were screened for ambiguity in the sequences (maximum ambiguity allowed: 0) and for homopolymers (maximum homopolymer length allowed: 8 nt). Chimera detection was done by using the algorithm Uchime (Edgar et al., 2011) which allows for a fast, sensitive and accurate detection of chimera. Sequences were then clustered as OTU (using the threshold of 3% of divergence) and OTU representative sequences were then classified at the taxonomic levels by comparisons with sequences from a self-made unified database containing the SILVA, Ribosomal Database project and HOMD database (Bootstrap cutoff of 80). Species identification was only possible when the best match in the database was from the HOMD therefore other sequences were assigned as unknown species (sp.).

### Statistical Analysis

An OTU table was used to calculate descriptive indices as alpha-diversity (non-parametric Shannon index), richness (Chao1 richness estimate, number of OTUs observed), evenness (non-parametric Shannon index-based measure of evenness) and dominance (Bergerparker-Index). Beta-diversity was assessed by calculating distance matrices based on Bray–Curtis distances. As inter-individual variation was important in our data, we assigned samples to ecotypes using a probabilistic modeling based on *k*-means algorithm (number of iterations 1,000,000) to cluster microbial communities into the major meta-communities. Clustering was confirmed and visualized by a heat-map and PCoA. The optimal number of clusters was defined by gap statistic (Tibshirani et al., 2001). A PERMANOVA adjusted for age and gender was performed to assess the statistical significance of differences in explanatory variables among samples or groups of samples. A LDA effect size (LEfSe) analysis was also performed to detect differentially abundant OTUs between groups. To compare the proportion of diseased patients between two or more groups, a multivariate analysis with a logistic regression model was performed adjusted for age and gender. For multiple groups’ comparison, a *post hoc* Tukey test was done. All statistical analysis were performed with MOTHUR 1.33.0 (Schloss et al., 2009) and R 3.1.2 (R Development Core Team, 2014) [package: gplots (Warnes et al., 2009), vegan (Dixon, 2003), multcomp (Hothorn et al., 2008)].

## Results

### Identification of Two Major Microbial Ecotypes in a Population-Wide Study

**FIGURE 1:**
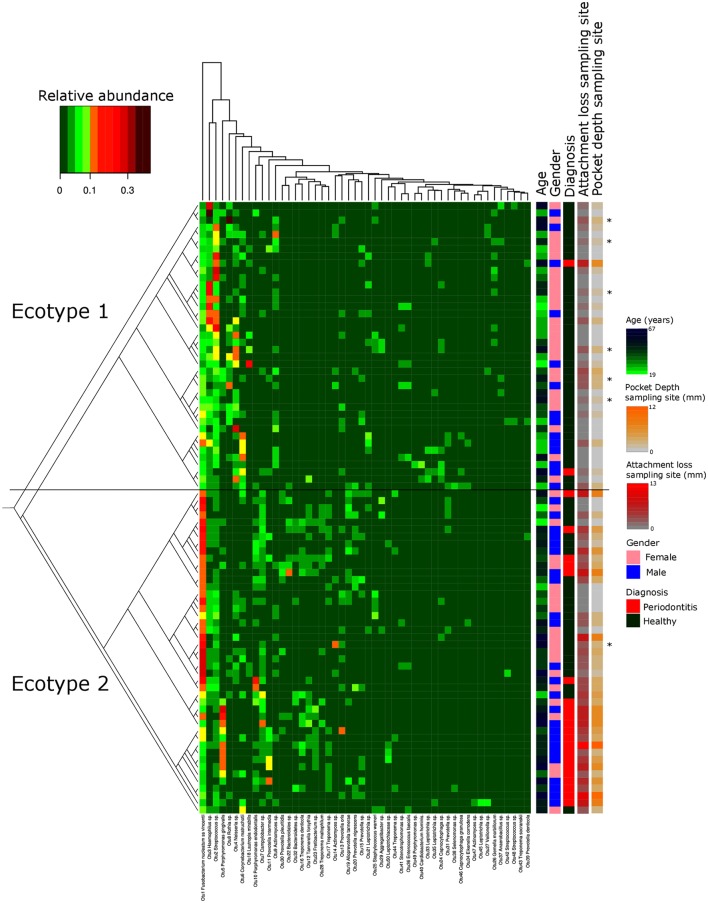
**Microbiota’s structure analysis of the subgingival communities in periodontitis and correlation of microbial profiles with clinical and demographic measurements.** Shown is a heatmap of the relative abundance for the top 50 OTUs found in the study cohort. Patients are in rows and OTUs are in columns. The color key is shown on the upper left. Patients are clustered based on the Bray–Curtis distance between their microbial communities validated by a *k*-mean algorithm probabilistic modeling (1000000 iterations). These distances are represented in the left tree showing two distinct clusters (Ecotypes 1 and 2). Relevant clinical and demographic characteristics are depicted in color codes at the right side. Patients diagnosed for periodontitis via the full mouth diagnosis but sampled at a non-diseased local site are symbolized by a ^∗^.

**FIGURE 2:**
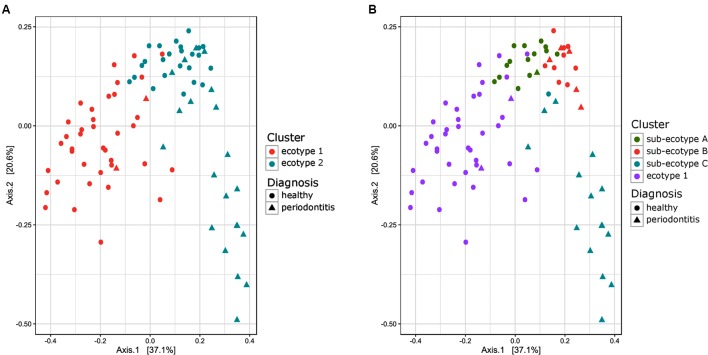
**Principal Coordinate Analysis plot comparing Bray–Curtis distance between the samples of the subgingival communities in periodontitis.** **(A)** Samples are color-coded based on a two clusters model (*k* = 2). **(B)** Samples are color coded based on a four clusters model (*k* = 4). The shape of the dots represents the diagnosis of the patient: triangle for healthy/mild periodontitis patient and circle for moderate/severe periodontitis patients.

Thirty-eight males and 47 females were enrolled in this study. The mean age of all participants was 42.6 years (y) (range19–67 years). Twenty-nine Resettlers from the former Soviet Union (34.1%), 17 Germans (20%), and 39 Turks (45.9%) participated in this study. Based on full mouth diagnosis according to the CDC AAP definition 30 (23+7) subjects (35.3%) were diagnosed with moderate to severe periodontitis, which is in the range of published representative studies (Eke et al., 2012). However, samples from diseased teeth were only obtained from 23 subjects whereas in 7 subjects with full mouth diagnosis “moderate/severe periodontitis” sampling was only done at a non-diseased site. As these sites are probably containing a different microbiome (Griffen et al., 2012; Shi et al., 2015) they were reclassified as healthy/mild periodontitis according to the local disease status. From 2,431,538 reads that we obtained from all 85 subjects, we cleaned and removed chimera to end up with 2,248,918 clean non-chimeric reads. We sub-sampled 4,218 reads per samples and validated sufficient coverage using rarefaction curves and calculating the Good’s estimator of coverage [mean coverage: 97.8% (min–max: 95.6–99.0%)]. A total of 3,273 OTUs were found. The mean numbers of OTUs per sample was 213 (min–max: 139–332). To analyze the cohort we estimated major clustering of our samples using a *k*-means algorithm based on a Bray–Curtis distance matrix. This algorithm indicated that the study population clustered into two main clusters (ecotype 1: 47%; ecotype 2: 53%) (**Figures 1**, **2A**), which were significantly divergent (PERMANOVA adjusted for age and gender: *R*^2^-value 0.26778, *p*-value < 0.001). Samples from the ecotype 1 showed a significantly richer microbiota (number of OTUs observed) and a lower biomass (estimated by the number of copies of the 16S rRNA gene by qPCR). No other differences in the alpha diversity were detected (**Table 1**).

**Table 1:** Microbiological and clinical differences between the ecotypes 1 and 2.

	Ecotype 1	Ecotype 2	*P*-value
Number of 16S rRNA gene copies	116519	653924	3.57E-03
log10 (number of 16S rRNA gene copies)	5.07	5.81	9.76E-04
Number of OTUs/samples	229	199	0.00674
Chao estimate	410.1	359.2	0.07537
Non-parametric Shannon index	3.583	3.566	0.74244
Evenness	0.6461541	0.6621499	0.23617
Bergerparker index	0.1935	0.1979	0.75601
Age (year)	38.2	46.2	0.00525
Pocket depth (mm)	2.6	4.2	3.99E-03
Attachment loss (mm)	1.5	4.2	6.33E-04
Gender (M/F)	14/26	24/21	0.07435
Periodontitis (Yes/No)	2/38	21/24	0.00391
Self reported periodontitis treatment (Yes/No/No answer)	4/35/1	15/28/2	0.80236

*This table summarizes the mean value of each ecotype for each parameter measured. Group comparisons were tested using a multivariate analysis with logistic regression with an adjustment for age and gender*.

### Microbial Ecotypes Are Associated with Periodontal Disease Status

**FIGURE 3:**
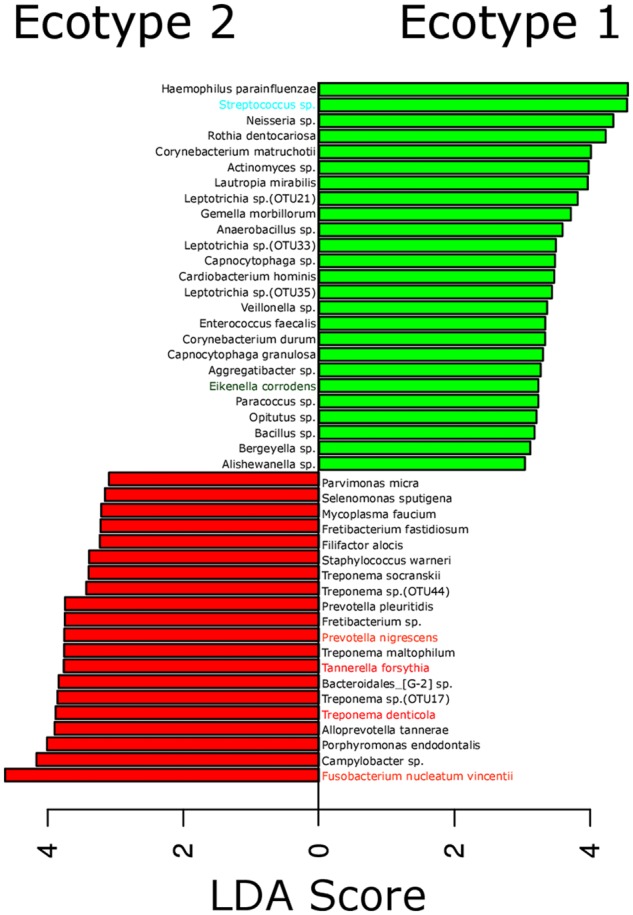
**Most differentially abundant OTUs between the microbial ecotypes 1 and 2.** Bar plot representing the most differentially abundant OTUs between the ecotypes 1 and 2 as detected by a LDA effect size (LEfSe) analysis (LDA score > 2). OTUs signature specific to the ecotypes 1 and 2 are, respectively, in green and red. OTUs are named by their taxonomic assignment and color coded depending of their membership to the red (in red), orange (in orange), green (in green), and yellow (in blue) complex.

The strong divergence between the microbial structures of the two ecotypes was explained by the high number of OTUs differentially abundant (87 OTUs) and the weight of those OTUs; 34 OTUs of the top 50 most abundant OTUs (accounting for 83.2% of the microbiota) were divergent between the two ecotypes (**Figure 3** and Supplementary Table S1). We noticed an increase in the ecotype 2 of four specific species (*Prevotella nigrescens, Fusobacterium nucleatum vincentii*, *Tannerella forsythia*, and *Treponema denticola*) known to be involved in periodontitis and a decrease of two species (*Eikenella corrodens* and *Streptococcus* sp.) known to belong to the healthy microbiota (Socransky et al., 1998). Of notice, the microbial clustering also reflected clinical measurements. Ecotype appears to be related to periodontitis: 2 out of 40 (5%) in ecotype 1 and 21 out of 45 (46.7%) in ecotype 2 showed diseased sampling sites. We used a generalized linear model (binomial family) to evaluate the accuracy of association between ecotype and disease status (accuracy: 72.9%, *p*-value < 0.001). The odds ratio for ecotype 2 estimated from a logistic regression model adjusted for age and sex is 11.0 (*p*-value = 0.00391) (**Figure 1**). This was reflected also by the current local inflammatory status at the sampling site, represented by ‘BOP’ (**Supplementary Figure S1**). Patients belonging to the ecotype 1 were younger and exhibited a lower PD and AL at the sample site (**Table 1**). No divergence of gender distribution was observed among the ecotypes and none of the other different clinical parameters showed a strong divergence.

### Microbial Ecotype 2 Shows Further Sub-clustering That Associates with Distinct Clinical Parameters

**FIGURE 4:**
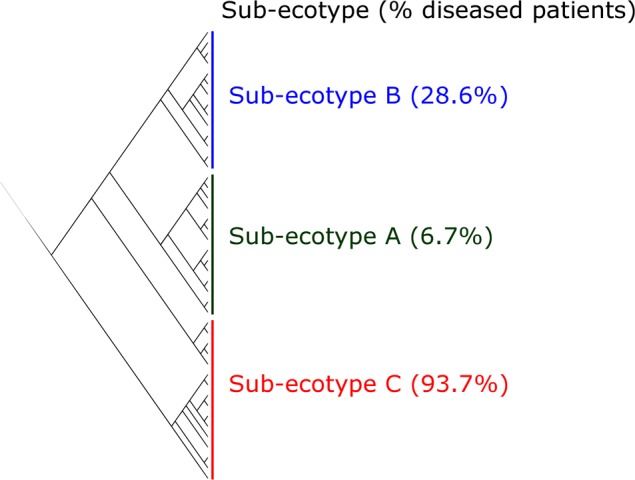
**Clustering of the ecotype 2 based on Bray–Curtis distances.** The ecotype 2 was clustered in three sub-ecotypes (A–B–C) based on *k*-means algorithm (1000000 iterations). One sample was alone in a fifth cluster and was removed. The optimal number of clusters was assessed with the gap statistic (**Supplementary Figure S2**). Indicated is the percentage of diseased patients in the respective sub-ecotypes.

Ecotype 2 was phenotypically more heterogeneous with an assembly of healthy participants, subjects with mild PD and AL as well as periodontitis patients, indicating a potential need of further clustering. This was also supported by the distribution on the PCoA plot and the dendrogram based on Bray–Curtis distances (**Figures 2B**, **4**). Using gap statistic (Tibshirani et al., 2001) (**Supplementary Figure S2**), the maximum number of reasonable clusters was estimated to be four clusters (corresponding to the ecotype 1 + three sub-ecotypes). According to this, three additional clusters (sub-ecotype A-B-C) within ecotype 2 could be identified. We again used a generalized linear model (binomial family) to evaluate the accuracy of association between ecotype and disease status (accuracy: 77.6%, *p*-value < 0.001). This finding was further confirmed by a PERMANOVA adjusted for age and gender, which showed an increase of the *R*^2^-value with the usage of four clusters (PERMANOVA Healthy vs. disease: *R*^2^-value = 0.44, *p*-value < 0.001). Furthermore the four ecotypes showed significantly different microbiota (Ecotype1 vs. sub-ecotype A: *R*^2^-value = 0.31, *p*-value < 0.01; Ecotype1 vs. sub-ecotype B: *R*^2^-value = 0.20, *p*-value < 0.01; Ecotype1 vs. sub-ecotype C: *R*^2^-value = 0.33, *p*-value < 0.01; sub-ecotype A vs. sub-ecotype B: *R*^2^-value = 0.19, *p*-value < 0.01; sub-ecotype A vs. sub-ecotype C: *R*^2^-value = 0.36, *p*-value < 0.01; sub-ecotype B vs. sub-ecotype C: *R*^2^-value = 0.42, *p*-value < 0.01).

**FIGURE 5:**
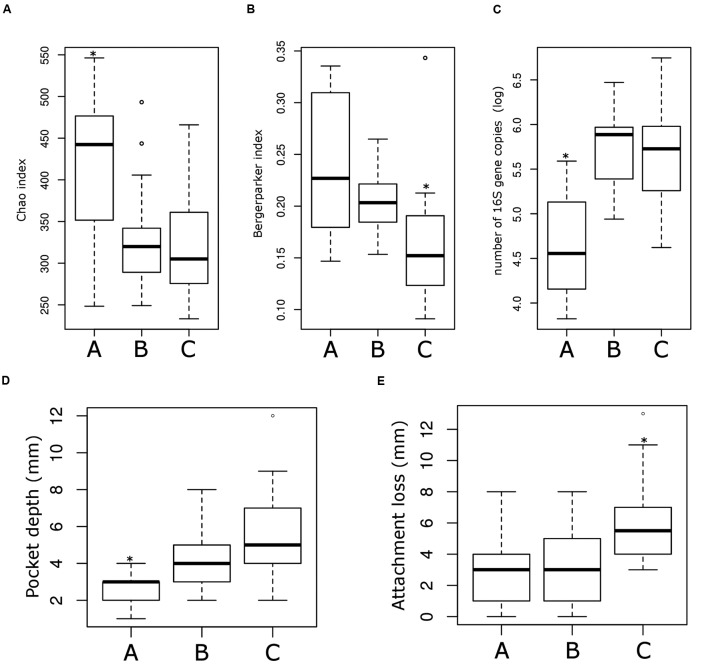
**Microbiological and clinical differences among the ecotype 2.** Bar plots showing the difference between the three sub-ecotypes A, B, and C regarding richness (Chao index) **(A)**, dominance (Bergerparker index) **(B)**, biomass (Log of the number of 16S rRNA gene copies) **(C)**, PD at the sampling site **(D)** and the AL at the sampling site **(E)**. Pairwise comparisons were tested using *t*-test or Wilcoxon test as appropriate with a Bonferroni correction; significance is indicated by a star (^∗^).

Sub-ecotype A contain one moderate/severe periodontitis-patient (6.7%) and was significantly richer than the two other sub-ecotypes (**Figure 5A**), with a higher dominance index (**Figure 5B**) and a lower biomass of bacteria (**Figure 5C**). Sub-ecotype A also included subjects with the lowest PD (**Figure 5D**) and the lowest proportion of BOP positive sites (**Supplementary Figure S1**). Therefore, samples from this sub-ecotype A are phenotypically closer related to the healthy samples from ecotype 1.

On the opposite, sub-ecotype C showed the highest PD, the highest AL, a low richness and a high biomass (**Figures 5D,E**). Sub-ecotype C also had the lowest dominance measured by the Bergerparker index, probably because of a consortium of several different pathogens replacing otherwise dominating single OTUs (**Figure 5B**). This sub-ecotype contained most of the moderate/severe periodontitis patients with increased AL (median 5.5 mm), PDs (median 5 mm).

Yet, sub-ecotype B showed an intermediate phenotype with a low richness associated to a high biomass and dominance. In the clinical measurements, sub-ecotype B showed a mild PD (median: 4 mm), moderate gingival bleeding (proportions 50% positive) and a low AL (median: 3 mm). The data can indicate that those samples might be from mild periodontitis, gingivitis or show a controlled situation in treated patients. Despite the fact that none of the other clinical measurements was significantly different among the three clusters, we observed in patients from the ecotype 2 a division from healthy carrier (without clinical signs of periodontitis but mild signs of gingival bleeding) (sub-ecotype A) to severely diseased patients (sub-ecotype C) with an intermediate stage (sub-ecotype B).

### Ecotype 2 Shows Heterogeneous Microbiota and a Clear Gradient of Distinct Pathogens

**FIGURE 6:**
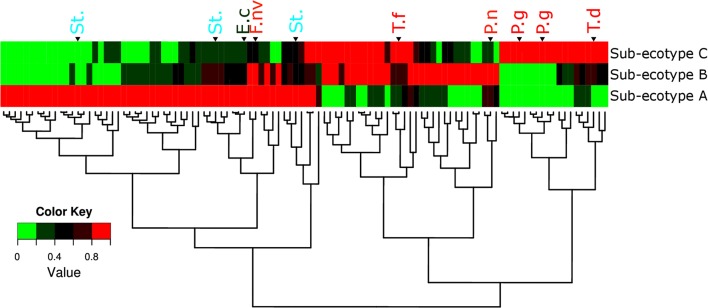
**Microbiota’s structure divergence between the sub-ecotype A, B and C.** Heatmap of the normalized abundance for the 106 OTUs differentially abundant between the three sub-ecotypes found by the LEfSe analysis. The abundance was normalized for each OTU by the maximal value observed among the sub-ecotypes to obtain a value ranking between 0 and 1. This normalization allows a good visualization of the differential abundance among the three clusters independently of the relative abundance. OTUs are in rows and clusters are in columns. The color key is shown on the lower left. The dendrograms show the relationship between the three sub-ecotypes based on the Bray–Curtis distance. OTUs are named by their taxonomic assignment and color coded depending of their membership to the red (in red), orange (in orange), green (in green), and yellow (in blue) complex define by Socransky. For an extensive view of all the OTUs, see the expanded version of this figure in **Supplementary Figure S3**. OTUs are clustered regarding their patterns of evolution among the three sub-ecotypes. P.g, *Porphyromonas gingivalis*; T.d, *Treponema denticola*; T.f, *Tannerella forsythia*; P.n, *Prevotella nigrescens*; St., *Streptococcus* sp.; F.nv, *Fusobacterium nucleatum vincentii*; E.c, *Eikenella corrodens*.

**FIGURE 7:**
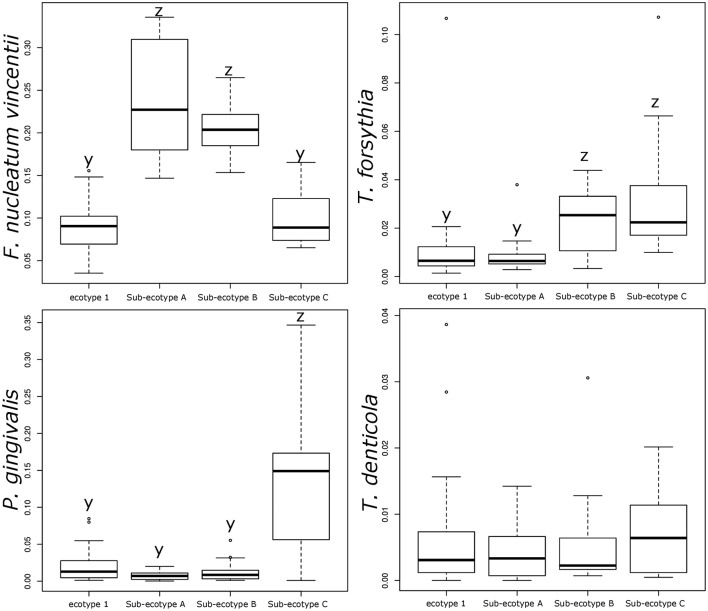
**Relative abundance of the classical bacteria *F. nucleatum vincentii, P. gingivalis, T. forsythia*, and *T. denticola* associated with periodontitis.** Bar plots showing the difference in relative abundance of the classical periodontitis pathogens between ecotype 1 and the three sub-ecotypes A, B, and C. Pairwise comparisons were tested using *t*-test or Wilcoxon test as appropriate with a Bonferroni correction. Mean with a different letter are significantly different (corrected *p*-value < 0.01).

Differentiation of microbial ecotype 2 into three clusters was suggestive for a gradual division of microbial changes between groups (Supplementary Table S2). We found 62 OTUs divergent between the sub-ecotype A and B (Bray–Curtis distance = 0.33), 29 OTUs between the sub-ecotype B and C (Bray–Curtis distance = 0.33) and 84 OTUs between the two extreme sub-ecotype A and C (Bray–Curtis distance = 0.51). Furthermore, most of the divergent OTUs showed a clear gradient pattern of abundance from sub-ecotype A to sub-ecotype B to sub-ecotype C (**Figure 6**). Based on Socranski definition of the key pathogens in periodontitis (Socransky et al., 1998), we observed that sub-ecotype A which mainly contained healthy people had higher relative abundances of the known “green complex” and “yellow complex” bacteria as well as one species from the “orange complex”; *F. nucleatum vincentii*, whereas the other “orange” and “red” complex bacteria showed up in sub-ecotype B and C, respectively (**Figure 6** and for a close-up **Supplementary Figure S3**). Specifically, we analyzed abundance of *P. gingivalis, T. forsythia, T. denticola*, and *F. nucleatum vincentii* (**Figure 7**). Whereas *P. gingivalis* was not overall different between Ecotypes 1 and 2 (**Figure 3**) it turned out that within the ecotype 2 there was a clear and significant evolution: *P. gingivalis* increased significantly in abundance in sub-ecotype C where it became highly abundant (up to 35%). Of note, ecotype 1 and sub-ecotype A and B contained *P. gingivalis*, albeit at low abundance. A likewise trend (not significant) was seen for *T. denticola*. *T. forsythia* showed an increase in sub-ecotype B and then further in sub-ecotype C.

**FIGURE 8:**
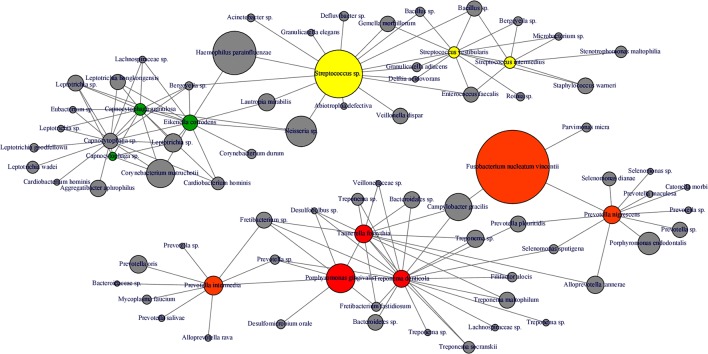
**Microbial co-occurrence network from subgingival microbiota.** Co-occurrence networks were constructed based on the relative abundance of the most abundant OTUs (Relative abundance > 1%). Each vertex represent an OTUs and is color coded based on the bacterial complex characterization by Socransky et al. (1998). The gray vertices represent OTUs that were not classified by Socransky et al. (1998). In order to lighten the network, only direct correlation between bacteria known to be associated with periodontitis complexes are displayed. The size of the vertex is proportional to the average relative abundances of the OTUs in the whole dataset. Each edge represents a positive correlation between the two OTUs with a Spearman’s correlation coefficient > 0.4.

Interestingly, abundance of *F. nucleatum* was one of the decisive discriminators that differentiated healthy person in ecotype 1 from healthy subjects from the ecotype 2 (Sub-ecotype B). *F. nucleatum* declined from sub-ecotype A to B to C in abundance. Healthy patients from the ecotype 1 do not carry classical pathogens at high level of abundance. *F. nucleatum* increases greatly in early stages of the disease (Sub-ecotype A, minor PD and bone loss). In the second stage (Sub-ecotype B, mild PD and reduced bone loss), *T. forsythia* increases significantly and remains at this abundance while *P. gingivalis* becomes the dominant species during the severe stage (Sub-ecotype C, severe PD and bone loss). Therefore, we clearly observe an ecological clustering among those classical pathogens associated with the increased severity of the disease. Those classical key pathogens also co-occur with several other bacteria, which are not yet classified as associated with periodontitis (**Figure 8**). This global co-occurrence pattern highlights two major networks; one associated to the abundance of bacteria from the yellow and green complexes while the other is characterized by the abundance of bacteria from the orange and red complexes (**Figure 8**).

## Discussion

A recent concept in the field of periodontitis states that a succession of keystone pathogens can disrupt tissue homeostasis and changes the composition of the commensal microbiota thereby generating host immune modulation and dysbiosis that is responsible for periodontitis (Hajishengallis et al., 2011, 2012). In this study, we observed two different microbiological ecotypes derived from sub-gingival samples within a population-based study; one major ecotype was homogeneously composed of healthy or only mild periodontitis subjects and the other more heterogeneous ecotype consisted of a mixture of three distinct clusters (sub-ecotypes A, B, and C). To our knowledge this is the first study of a population-based sample using NGS and showing a clear division in the microbiotas associated to the whole range of phenotypical variation in the disease from healthy to severe periodontitis.

In another study of 34 subjects with only periodontitis patients, two clusters could be found (Hong et al., 2015). In this study, one cluster was composed of bacteria known as being keystone pathogens (*P. gingivalis, T. forsythia*, and *T. denticola)* while the other cluster was composed of bacteria known as first colonizer from the orange complex (*Fusobacterium* sp.*, Prevotella* sp., and *Campylobacter* sp.) as well as bacteria from health-associated species. In a similar study by Park et al. (2015), 32 individuals have been allocated to three different groups (healthy, gingivitis, and periodontitis) according to clinical parameters. The healthy group dominated by *Streptococcus* sp., the gingivitis group was composed mostly by the genera *Streptococcus*, *Capnocytophaga*, *Haemophilus*, and *Leptotrichia* and the periodontitis group with a high abundance of *Porphyromonas*. Our findings match very well with those results as our sub-ecotype A and B together compared to the sub-ecotype C display the same contrast as the one showed by Hong et al. (2015). Also, the three clusters depicted by Park et al. (2015) seem to be reflected by our cohorts from the ecotype 1 (healthy cohort), the sub-ecotypes A and B (mild disease, gingivitis) and the sub-ecotype C (periodontitis). However, in our study we were able to depict four distinct groups correlating with periodontally healthy or mild disease and moderate or severe periodontitis. This resolution is probably due to the fact that the clustering was based on a population design and only on the microbiota profiles of 85 patients without pre-selection based on the clinical parameters.

The four clusters (ecotype 1 + three sub-ecotypes A–C) observed in our study are nevertheless associated with distinct clinical conditions. Those clinical conditions could be ordered based on the known process of the disease from healthy to severely diseased. Patients from the ecotype 1 are mostly healthy and younger individuals with shallow pockets and nearly no AL at the sample site and low BOP. Those patients are also characterized by a low abundance of classical pathogens (**Figures 1**, **7**) as shown in a previous study comparing the microbiota of periodontal compromised and matched healthy subjects (Griffen et al., 2012). Those results indicate the presence of a “healthy core microbiota” potentially linked to resistance against periodontitis. This concept of a “healthy core microbiota” was suggested in several studies before, which showed a high diversity and similarity to the different ecological niches within the oral cavity (Zaura et al., 2009; Bik et al., 2010). The sub-ecotype A from the ecotype 2 also displays shallow pockets, slightly higher BOP and minimal AL at the sample site with only healthy or mild periodontitis patients in that cluster (**Figures 3**, **4**). However, the microbiota undergo microbial colonization by *F. nucleatum vincentii* and first colonizers from the green and yellow complex indicating a new stage in the disease progression (Socransky et al., 1998; Kolenbrander et al., 2010). This result also supports the hypothesis that green complex is correlated to better health and that orange and yellow complexes are indicators of a first colonization leading to the establishment of the disease (Socransky et al., 1998; Kolenbrander et al., 2010). It also appears that yellow complex and green complex are co-occurring with species with yet unknown functions in the context of periodontitis during the early stage of the disease (**Figure 7**). On the other hand, *F. nucleatum* and *Prevotella* sp. seem to have a particular role as a bridging-organism between early and late colonizers (Socransky et al., 1998; Kolenbrander et al., 2010; Kirst et al., 2015). However, we identify additional bacteria that differ between those clusters and are directly co-occurring with this complex thus extending the classical complex concept (**Figure 8**).

The sub-ecotype B showed an increased PD but still a low AL reflecting an increase in the severity and higher BOP. The microbiota from this cluster is characterized by a high abundance of *F. nucleatum vincentii* like in the sub-ecotype A but also an increase of bacteria from the orange and red complex (*T. forsythia*, *P. nigrescens*) (Socransky et al., 1998) as well as many other species with unknown function (**Figure 6** and **Supplementary Figure S3**, Table S2). Finally, the sub-ecotype C clinically reflects the most severe stage of the disease with deep pockets, frequent BOP and high AL; those changes go along with further alterations in the microbiota, notably an increase of species from the red complex (e.g., *P. gingivalis* and *T. denticola*) (Socransky et al., 1998) and numerous other species not known so far to be associated to the disease (**Figure 8**). The latter might indicate a possible pathogenic role or a hitch hiking effect (**Figure 6** and **Supplementary Figure S3**, Table S2).

This study classified subjects primarily by the microbial ecotype, which was then associated to clinical signs of periodontitis. Periodontitis was classified by the CDC-AAP classification, which takes AL as well as PD into account. Thus it is a more specific definition as the widely used CPITN-index, which only uses maximal PD (Cutress et al., 1987). A periodontitis patient can have different stages of inflammation on different teeth, which might also be represented by intra-individual microbiome differences (Shi et al., 2015). We took only one sample from each patient from one randomized sampling site. In many other studies different sites were pooled, which might lead to mixing different microbial stages together. Our approach also has its drawbacks, because sites can be randomly selected from a patient classified as periodontitis, but with no apparent signs of inflammation or AL. Future studies should consider different inflammatory conditions by taking multiple samples at different stages of disease in one subject to account for these intra-individual differences.

## Conclusion

In this study, we observed the emergence of four discrete microbial communities within our population associated with clinical outcome of periodontitis ranging from healthy/mild periodontitis to moderate/severe periodontitis. The evolution of the abundance of the known pathogens involved in the disease as well as the increase of the severity parameters (AL, PD, and BOP) along the four ecotypes tend to prove a gradual change of microbiota during disease. Therefore, all together, our findings are supporting the concept of induced dysbiosis or polymicrobial synergy (Hajishengallis and Lamont, 2012). We propose a scenario of gradual change in subgingival microbiota, which goes along with clinical disease symptoms. Only in “end-stage” dysbiosis classical red complex bacteria gain overweight. Such a concept of induced dysbiosis including early, low abundant keystone pathogens has been suggested and supported by others before (Hajishengallis and Lamont, 2012; Hajishengallis et al., 2012; Park et al., 2015) but is now underlined by our findings in a population-based study. This scenario needs to be confirmed by future studies based on longitudinal design to validate the microbial variation over the different clusters in time. However, the findings in this study can already lead to an establishment of a microbial risk profile for clinically healthy patients.

## Ethics Statement

The ethical board of the medical faculty of Heidelberg (S-108/2011) had approved this study and all participants gave their written informed consent.

## Availability of Data and Materials

Clean sequences, metadata files and R script used for analysis have been uploaded, and are publicly available in Figshare (https://figshare.com/s/8da5157b93740ae58b8e).

## Author Contributions

SB, DH, HZ, NE, TH, HG, HB, T-SK, and AD made substantial contributions to conception and design. SB, DH, HZ, HB, NE, T-SK, and AD contribute to the acquisition of data, analysis and interpretation of data. SB, DH, HZ, NE, TH, HG, HB, T-SK, and AD have been involved in drafting the manuscript or revising it critically for important intellectual content. SB, DH, HZ, NE, TH, HG, HB, T-SK, and AD gave a final approval of the version to be published.

## Conflict of Interest Statement

The authors declare that the research was conducted in the absence of any commercial or financial relationships that could be construed as a potential conflict of interest.

## Supplementary Material

FIGURE S1 **Proportions of bleeding on probing (BOP) in each sub-cohort**. BOP was measured as presence or absence of bleeding on probing of the periodontal pocket. Statistical difference in BOP was calculated by a Tukey test based on a logistic regression. Column with a different letter are significantly different (corrected *p*-value < 0.01).

FIGURE S2 **Estimation of the optimal number of clusters in the population**. The number of clusters in our dataset was estimated via the gap statistic as described by Tibshirani et al. (2001).

FIGURE S3 **Microbiota’s structure divergence between the sub-ecotypes A, B, and C (expanded version)**.

## Abbreviations

AL: attachment loss
BOP: bleeding on probing
HOMD: human oral microbiome database
LDA: linear discriminant analysis
LEfSe: linear discriminant analysis effect size
NAKO: German National Cohort
NGS: next generation sequencing
OTU: operational taxonomic units
PCoA: principal coordinates analysis
PD: pocket depth
qPCR: quantitative PCR
