# Supplementary figures and images for: Clustering of Subgingival Microbiota Reveals Microbial Disease Ecotypes Associated with Clinical Stages of Periodontitis in a Cross-Sectional Study
Source: Front Microbiol. 2017 Mar 1;8:340. doi: 10.3389/fmicb.2017.00340 (PMC5331054; doi:10.3389/fmicb.2017.00340)

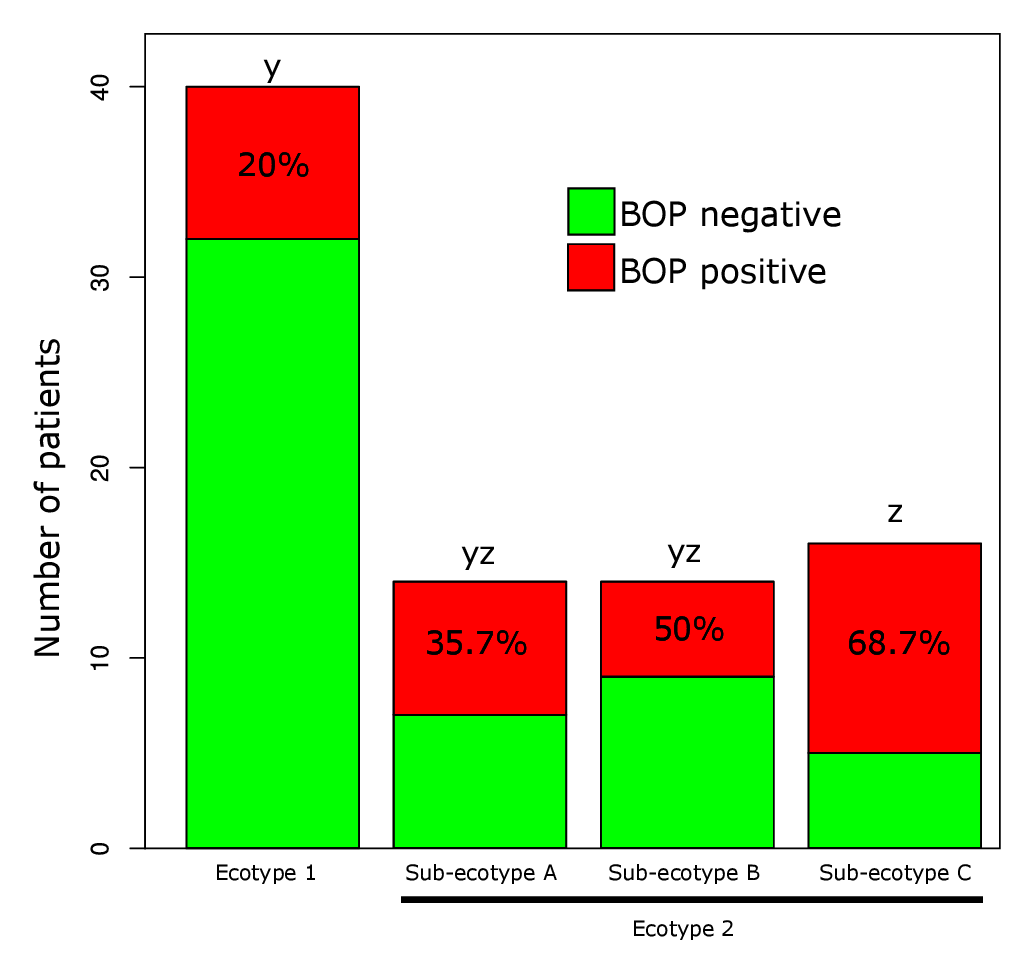

Supplement: FIGURE S1 — Proportions of bleeding on probing (BOP) in each sub-cohort. BOP was measured as presence or absence of bleeding on probing of the periodontal pocket. Statistical difference in BOP was calculated by a Tukey test based on a logistic regression. Column with a different letter are significantly different (corrected p-value < 0.01). [file Image_1.TIFF]

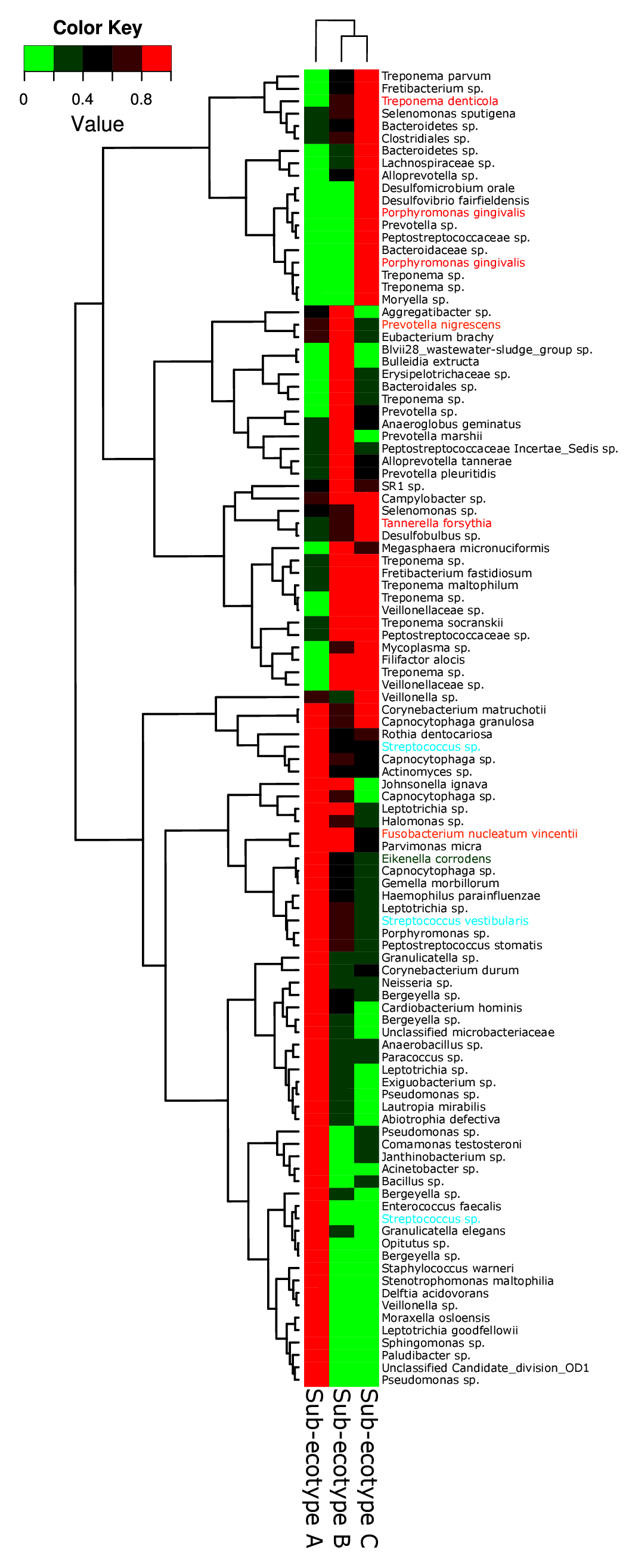

Supplement: FIGURE S3 — Microbiota’s structure divergence between the sub-ecotypes A, B, and C (expanded version). [file Image_3.TIFF]
