# Supplementary material for: Clustering of Subgingival Microbiota Reveals Microbial Disease Ecotypes Associated with Clinical Stages of Periodontitis in a Cross-Sectional Study
Source: Front Microbiol. 2017 Mar 1;8:340. doi: 10.3389/fmicb.2017.00340 (PMC5331054; doi:10.3389/fmicb.2017.00340)
Supplement: Supplementary file 4 [file Table_1.DOCX]

**Supplementary Table 1**

**Differentially abundant OTUs between the ecotype 1 and 2 detected by a linear discriminant analysis (LDA) effect size (LEfSe) analysis.**

This table shows all the OTUs detected as significantly differentially abundant and their taxonomic assignment and the corresponding bootstrap value (in brackets).No bootstrap values indicate that no confident match (cutoff 80) was found and therefore classification stops at the previous level.

| **Otu** | **Most abundant in** | **LDA Score** | **P-Value** | **taxonomy** |
| --- | --- | --- | --- | --- |
| 00001 | Ecotype_2 | 4.62987 | 4.6742E-08 | Bacteria(100);Fusobacteria(100);Fusobacteria(100);Fusobacteriales(100);Fusobacteriaceae(100);Fusobacterium(100);nucleatum_ss_vincentii_oral_taxon_200(100); |
| 00002 | Ecotype_1 | 4.54533 | 2.0769E-09 | Bacteria(100);Firmicutes(100);Bacilli(100);Lactobacillales(100);Streptococcaceae(100);Streptococcus(100);sp._oral_taxon_058(100); |
| 00003 | Ecotype_1 | 4.5569 | 8.0151E-07 | Bacteria(100);Proteobacteria(100);Gammaproteobacteria(100);Pasteurellales(100);Pasteurellaceae(100);Haemophilus(100);parainfluenzae_oral_taxon_718(84); |
| 00004 | Ecotype_1 | 4.34139 | 5.1978E-07 | Bacteria(100);Proteobacteria(100);Betaproteobacteria(100);Neisseriales(100);Neisseriaceae(100);Neisseria(100);sp. |
| 00006 | Ecotype_1 | 4.01408 | 0.00988116 | Bacteria(100);Actinobacteria(100);Actinobacteria(100);Actinomycetales(100);Corynebacteriaceae(100);Corynebacterium(100);matruchotii_oral_taxon_666(96); |
| 00007 | Ecotype_2 | 4.16747 | 1.0872E-10 | Bacteria(100);Proteobacteria(100);Epsilonproteobacteria(100);Campylobacterales(100);Campylobacteraceae(100);Campylobacter(100);sp. |
| 00008 | Ecotype_1 | 4.22869 | 0.00141325 | Bacteria(100);Actinobacteria(100);Actinobacteria(100);Actinomycetales(100);Micrococcaceae(100);Rothia(100);dentocariosa_oral_taxon_587(94); |
| 00009 | Ecotype_1 | 3.98087 | 0.00064438 | Bacteria(100);Actinobacteria(100);Actinobacteria(100);Actinomycetales(100);Actinomycetaceae(100);Actinomyces(100);sp._oral_taxon_171(100); |
| 00010 | Ecotype_2 | 4.01195 | 4.0673E-05 | Bacteria(100);Bacteroidetes(100);Bacteroides(100);Bacteroidales(100);Porphyromonadaceae(100);Porphyromonas(100);endodontalis_oral_taxon_273(100); |
| 00012 | Ecotype_2 | 3.76449 | 6.7587E-05 | Bacteria(100);Bacteroidetes(100);Bacteroides(100);Bacteroidales(100);Porphyromonadaceae(100);Tannerella(100);forsythia_oral_taxon_613(100); |
| 00016 | Ecotype_2 | 3.88698 | 0.00031553 | Bacteria(100);Spirochaetes(100);Spirochaetes(100);Spirochaetales(100);Spirochaetaceae(100);Treponema(100);denticola_oral_taxon_584(100); |
| 00017 | Ecotype_2 | 3.85885 | 1.2544E-05 | Bacteria(100);Spirochaetes(100);Spirochaetes(100);Spirochaetales(100);Spirochaetaceae(100);Treponema(100);sp. |
| 00018 | Ecotype_1 | 3.96742 | 6.8362E-06 | Bacteria(100);Proteobacteria(100);Betaproteobacteria(100);Burkholderiales(100);Burkholderiaceae(100);Lautropia(100);mirabilis_oral_taxon_022(100); |
| 00019 | Ecotype_2 | 3.89977 | 0.00058281 | Bacteria(100);Bacteroidetes(100);Bacteroides(100);Bacteroidales(100);Prevotellaceae(100);Alloprevotella(100);tannerae_oral_taxon_466(100); |
| 00020 | Ecotype_2 | 3.75608 | 0.0110604 | Bacteria(100);Bacteroidetes(100);Bacteroides(100);Bacteroidales(100);Prevotellaceae(100);Prevotella(100);nigrescens_oral_taxon_693(100); |
| 00021 | Ecotype_1 | 3.81676 | 0.00208424 | Bacteria(100);Fusobacteria(100);Fusobacteria(100);Fusobacteriales(100);Leptotrichiaceae(100);Leptotrichia(100);sp. |
| 00022 | Ecotype_2 | 3.84021 | 0.0180237 | Bacteria(100);Bacteroidetes(100);Bacteroides(100);Bacteroidales(100);Bacteroidales_[F-2](100);Bacteroidales_[G-2](100);sp._oral_taxon_274(100); |
| 00023 | Ecotype_2 | 3.75012 | 0.00044686 | Bacteria(100);Synergistetes(100);Synergistetes_[C-1](100);Synergistetes_[O-1](100);Synergistetes_[F-2](100);Fretibacterium(100);sp._oral_taxon_361(100); |
| 00024 | Ecotype_1 | 3.48076 | 0.023874 | Bacteria(100);Bacteroidetes(100);Flavobacteria(100);Flavobacteriales(100);Flavobacteriaceae(100);Capnocytophaga(100);sp. |
| 00025 | Ecotype_2 | 3.3911 | 0.0390686 | Bacteria(100);Firmicutes(100);Bacilli(100);Bacillales(100);Staphylococcaceae(100);Staphylococcus(100);warneri_oral_taxon_076(100); |
| 00026 | Ecotype_1 | 3.71575 | 4.6413E-06 | Bacteria(100);Firmicutes(100);Bacilli(100);Bacillales(100);Staphylococcaceae(100);Gemella(100);morbillorum_oral_taxon_046(100); |
| 00027 | Ecotype_1 | 3.36605 | 0.0064165 | Bacteria(100);Firmicutes(100);Clostridia(100);Clostridiales(100);Veillonellaceae(100);Veillonella(100);sp. |
| 00028 | Ecotype_2 | 3.75953 | 4.6091E-05 | Bacteria(100);Spirochaetes(100);Spirochaetes(100);Spirochaetales(100);Spirochaetaceae(100);Treponema(100);maltophilum_oral_taxon_664(100); |
| 00029 | Ecotype_1 | 3.27117 | 0.0169492 | Bacteria(100);Proteobacteria(100);Gammaproteobacteria(100);Pasteurellales(100);Pasteurellaceae(100);Aggregatibacter(97);sp. |
| 00030 | Ecotype_2 | 3.74693 | 0.00424484 | Bacteria(100);Bacteroidetes(100);Bacteroides(100);Bacteroidales(100);Prevotellaceae(100);Prevotella(100);pleuritidis_[NV]_oral_taxon_303(99); |
| 00033 | Ecotype_1 | 3.4952 | 0.00320355 | Bacteria(100);Fusobacteria(100);Fusobacteria(100);Fusobacteriales(100);Leptotrichiaceae(100);Leptotrichia(100);sp. |
| 00034 | Ecotype_1 | 3.23617 | 0.00028878 | Bacteria(100);Proteobacteria(100);Betaproteobacteria(100);Neisseriales(100);Neisseriaceae(100);Eikenella(99);corrodens_oral_taxon_577(99); |
| 00035 | Ecotype_1 | 3.43586 | 0.00077641 | Bacteria(100);Fusobacteria(100);Fusobacteria(100);Fusobacteriales(100);Leptotrichiaceae(100);Leptotrichia(100);sp. |
| 00036 | Ecotype_1 | 3.33895 | 0.00029796 | Bacteria(100);Firmicutes(100);Bacilli(100);Lactobacillales(100);Enterococcaceae(100);Enterococcus(100);faecalis_oral_taxon_604(100); |
| 00037 | Ecotype_1 | 3.59113 | 1.1882E-05 | Bacteria(100);Firmicutes(100);Bacilli(100);Bacillales(98);Bacillaceae(97);Anaerobacillus(94);sp. |
| 00040 | Ecotype_1 | 3.46946 | 0.00026301 | Bacteria(100);Proteobacteria(100);Gammaproteobacteria(100);Cardiobacteriales(100);Cardiobacteriaceae(100);Cardiobacterium(100);hominis_oral_taxon_633(100); |
| 00043 | Ecotype_2 | 3.39701 | 1.3956E-06 | Bacteria(100);Spirochaetes(100);Spirochaetes(100);Spirochaetales(100);Spirochaetaceae(100);Treponema(100);socranskii_oral_taxon_769(96); |
| 00044 | Ecotype_2 | 3.4329 | 0.00464529 | Bacteria(100);Spirochaetes(100);Spirochaetes(100);Spirochaetales(100);Spirochaetaceae(100);Treponema(100);sp. |
| 00046 | Ecotype_1 | 3.30413 | 0.0039496 | Bacteria(100);Bacteroidetes(100);Flavobacteria(100);Flavobacteriales(100);Flavobacteriaceae(100);Capnocytophaga(100);granulosa_oral_taxon_325(100); |
| 00051 | Ecotype_1 | 3.33644 | 8.5399E-05 | Bacteria(100);Actinobacteria(100);Actinobacteria(100);Actinomycetales(100);Corynebacteriaceae(100);Corynebacterium(100);durum_oral_taxon_595(100); |
| 00052 | Ecotype_2 | 3.1585 | 0.00188704 | Bacteria(100);Firmicutes(100);Clostridia(100);Clostridiales(100);Veillonellaceae(100);Selenomonas(100);sputigena_oral_taxon_151(100); |
| 00054 | Ecotype_1 | 3.1787 | 0.00030274 | Bacteria(100);Firmicutes(100);Bacilli(100);Bacillales(100);Bacillaceae(100);Bacillus(99);sp. |
| 00055 | Ecotype_1 | 3.23406 | 5.6584E-05 | Bacteria(100);Proteobacteria(100);Alphaproteobacteria(100);Rhodobacterales(100);Rhodobacteraceae(100);Paracoccus(100);sp. |
| 00056 | Ecotype_2 | 3.23316 | 0.00046368 | Bacteria(100);Firmicutes(100);Clostridia(100);Clostridiales(100);Peptostreptococcaceae_[XI](100);Filifactor(100);alocis_oral_taxon_539(100); |
| 00059 | Ecotype_2 | 3.21258 | 0.0142621 | Bacteria(100);Tenericutes(100);Mollicutes(100);Mycoplasmatales(100);Mycoplasmataceae(100);Mycoplasma(100);faucium_oral_taxon_606(89); |
| 00060 | Ecotype_1 | 3.2094 | 0.00117137 | Bacteria(100);Verrucomicrobia(100);Opitutae(100);Opitutales(96);Opitutaceae(96);Opitutus(87);sp. |
| 00062 | Ecotype_2 | 3.09782 | 0.0040235 | Bacteria(100);Firmicutes(100);Clostridia(100);Clostridiales(100);Peptostreptococcaceae_[XIII](100);Parvimonas(100);micra_oral_taxon_111(93); |
| 00063 | Ecotype_2 | 3.22313 | 2.3194E-05 | Bacteria(100);Synergistetes(100);Synergistetes_[C-1](100);Synergistetes_[O-1](100);Synergistetes_[F-2](100);Fretibacterium(100);fastidiosum_oral_taxon_363(100); |
| 00064 | Ecotype_1 | 2.94207 | 6.0728E-05 | Bacteria(100);Firmicutes(100);Bacilli(100);Lactobacillales(100);Carnobacteriaceae(100);Granulicatella(100);sp. |
| 00066 | Ecotype_1 | 2.96994 | 0.00229087 | Bacteria(100);Actinobacteria(100);Actinobacteria(100);Actinomycetales(100);Micrococcaceae(96);Rothia(95);sp. |
| 00071 | Ecotype_1 | 2.99818 | 0.0233928 | Bacteria(100);Proteobacteria(100);Betaproteobacteria(100);Burkholderiales(100);Comamonadaceae(100);Ottowia(100);sp._oral_taxon_894(100); |
| 00072 | Ecotype_1 | 3.03903 | 0.00375791 | Bacteria(100);Proteobacteria(100);Gammaproteobacteria(100);Alteromonadales(100);Alteromonadaceae(100);Alishewanella(100);sp. |
| 00073 | Ecotype_1 | 2.62269 | 0.00038063 | Bacteria(100);Bacteroidetes(100);Bacteroides(100);Bacteroidales(100);Prevotellaceae(100);Prevotella(100);sp._oral_taxon_472(91); |
| 00074 | Ecotype_1 | 3.11871 | 0.00018694 | Bacteria(100);Bacteroidetes(100);Flavobacteria(100);Flavobacteriales(100);Flavobacteriaceae(100);Bergeyella(100);sp._oral_taxon_322(100); |
| 00076 | Ecotype_1 | 2.79883 | 1.8828E-05 | Bacteria(100);Proteobacteria(100);Gammaproteobacteria(100);Pseudomonadales(100);Moraxellaceae(100);Acinetobacter(100);sp._oral_taxon_408(100); |
| 00079 | Ecotype_2 | 2.99208 | 0.0194336 | Bacteria(100);Firmicutes(100);Clostridia(100);Clostridiales(100);Veillonellaceae(100);Dialister(100);sp._oral_taxon_502(100); |
| 00080 | Ecotype_1 | 2.9208 | 6.2745E-05 | Bacteria(100);Firmicutes(100);Bacilli(100);Bacillales(100);Family_XII(100);Exiguobacterium(100);sp. |
| 00083 | Ecotype_2 | 2.86051 | 0.00585467 | Bacteria(100);Bacteroidetes(100);Bacteroides(100);Bacteroidales(100);Prevotellaceae(100);Prevotella(100);sp._oral_taxon_292(90); |
| 00084 | Ecotype_2 | 2.77722 | 0.0392407 | Bacteria(100);Bacteroidetes(100);Bacteroides(100);Bacteroidales(100);Prevotellaceae(100);Alloprevotella(100);sp._oral_taxon_912(100); |
| 00089 | Ecotype_1 | 2.58398 | 0.00418542 | Bacteria(100);Firmicutes(100);Bacilli(100);Lactobacillales(100);Carnobacteriaceae(100);Granulicatella(100);elegans_oral_taxon_596(92); |
| 00097 | Ecotype_1 | 2.66969 | 0.00172408 | Bacteria(100);Proteobacteria(100);Betaproteobacteria(100);Neisseriales(100);Neisseriaceae(100);Kingella(100);sp. |
| 00099 | Ecotype_2 | 2.49258 | 0.0304026 | Bacteria(100);Actinobacteria(100);Actinobacteria(100);Actinomycetales(100);Actinomycetaceae(100);Actinomyces(100);sp._oral_taxon_897(95); |
| 00104 | Ecotype_2 | 2.88876 | 0.00149726 | Bacteria(100);Firmicutes(100);Clostridia(100);Clostridiales(100);Veillonellaceae(100);Veillonellaceae_[G-1](100);sp._oral_taxon_129(99); |
| 00113 | Ecotype_2 | 2.74672 | 0.00028558 | Bacteria(100);Firmicutes(100);Clostridia(100);Clostridiales(100);Peptostreptococcaceae_[XI](100);Eubacterium_[XI][G-3](100);brachy_oral_taxon_557(100); |
| 00114 | Ecotype_1 | 2.80852 | 0.00050571 | Bacteria(100);Firmicutes(99);Erysipelotrichi(98);Erysipelotrichales(97);Erysipelotrichaceae(97);Erysipelothrix(73);sp. |
| 00115 | Ecotype_2 | 2.59333 | 0.0234362 | Bacteria(100);Firmicutes(100);Clostridia(100);Clostridiales(100);Veillonellaceae(100);Dialister(100);invisus_oral_taxon_118(100); |
| 00116 | Ecotype_2 | 2.78181 | 0.00211495 | Bacteria(100);Firmicutes(100);Clostridia(100);Clostridiales(100);Clostridiales_[F-1](100);Clostridiales_[F-1][G-1](100);sp._oral_taxon_093(100); |
| 00117 | Ecotype_2 | 2.78419 | 0.01016 | Bacteria(100);Proteobacteria(100);Deltaproteobacteria(100);Desulfobacterales(100);Desulfobulbaceae(100);Desulfobulbus(100);sp._oral_taxon_041(100); |
| 00119 | Ecotype_1 | 2.75861 | 2.8042E-05 | Bacteria(100);Firmicutes(100);Clostridia(100);Clostridiales(100);Clostridiaceae_2(100);unclassified(96);sp. |
| 00121 | Ecotype_1 | 2.71721 | 0.00289509 | Bacteria(100);Firmicutes(100);Bacilli(100);Lactobacillales(100);Carnobacteriaceae(100);Alkalibacterium(95);sp. |
| 00123 | Ecotype_2 | 2.69089 | 0.0100541 | Bacteria(100);Firmicutes(100);Clostridia(100);Clostridiales(100);Lachnospiraceae_[XIVa](100);Catonella(100);morbi_oral_taxon_165(100); |
| 00125 | Ecotype_1 | 2.69632 | 0.00787701 | Bacteria(100);Proteobacteria(100);Betaproteobacteria(100);Burkholderiales(100);Comomonadaceae(100);Delftia(100);acidovorans_oral_taxon_023(100); |
| 00128 | Ecotype_2 | 2.85129 | 1.5379E-05 | Bacteria(100);Firmicutes(100);Clostridia(100);Clostridiales(100);Veillonellaceae(100);Veillonellaceae_[G-1](100);sp._oral_taxon_155(100); |
| 00132 | Ecotype_1 | 2.74547 | 9.4348E-05 | Bacteria(100);Proteobacteria(100);Gammaproteobacteria(100);Oceanospirillales(100);Halomonadaceae(100);Halomonas(100);sp. |
| 00134 | Ecotype_1 | 2.60918 | 0.00051572 | Bacteria(100);Firmicutes(100);Clostridia(100);Clostridiales(100);Family_XII(100);Fusibacter(100);sp. |
| 00135 | Ecotype_1 | 2.66074 | 0.026984 | Bacteria(100);Bacteroidetes(100);Sphingobacteriia(100);Sphingobacteriales(100);Cyclobacteriaceae(100);Mongoliitalea(100);sp. |
| 00136 | Ecotype_2 | 2.73953 | 0.0429586 | Bacteria(100);Firmicutes(100);Clostridia(100);Clostridiales(100);Peptostreptococcaceae_[XI](100);Eubacterium_[XI][G-5](100);saphenum_oral_taxon_759(100); |
| 00141 | Ecotype_2 | 2.76295 | 0.00010932 | Bacteria(100);Firmicutes(100);Clostridia(100);Clostridiales(100);Family_XIII(98);unclassified(98);sp. |
| 00156 | Ecotype_2 | 2.72101 | 0.00016536 | Bacteria(100);Bacteroidetes(100);Bacteroidetes_[C-1](100);Bacteroidetes_[O-1](100);Bacteroidetes_[F-1](100);Bacteroidetes_[G-3](100);sp._oral_taxon_280(100); |
| 00157 | Ecotype_2 | 2.7383 | 0.00026663 | Bacteria(100);Firmicutes(100);Clostridia(100);Clostridiales(100);Peptostreptococcaceae_[XI](100);Mogibacterium(100);timidum_oral_taxon_042(89); |
| 00160 | Ecotype_2 | 2.51313 | 0.0324316 | Bacteria(100);Bacteroidetes(100);Bacteroides(100);Bacteroidales(100);Prevotellaceae(100);Prevotella(100);baroniae_oral_taxon_553(97); |
| 00161 | Ecotype_1 | 2.62037 | 0.00729832 | Bacteria(100);Proteobacteria(100);Gammaproteobacteria(100);Pseudomonadales(100);Pseudomonadaceae(100);Pseudomonas(100);sp. |
| 00162 | Ecotype_1 | 2.69176 | 0.0023604 | Bacteria(100);Actinobacteria(100);Actinobacteria(100);Actinomycetales(100);Micrococcaceae(82);Kocuria(70);sp. |
| 00163 | Ecotype_1 | 2.76456 | 0.00034222 | Bacteria(100);Proteobacteria(100);Betaproteobacteria(100);Burkholderiales(100);Oxalobacteraceae(100);Janthinobacterium(61);sp. |
| 00164 | Ecotype_2 | 2.71517 | 0.00018316 | Bacteria(100);Firmicutes(100);Clostridia(100);Clostridiales(100);Peptostreptococcaceae_[XI](100);Eubacterium_[XI][G-6](100);sp. |
| 00173 | Ecotype_1 | 2.79375 | 0.00059195 | Bacteria(100);Candidate_division_OD1(100);unclassified(100);unclassified(100);unclassified(100);unclassified(100);sp. |
| 00175 | Ecotype_1 | 2.7605 | 0.00065857 | Bacteria(100);Proteobacteria(100);Gammaproteobacteria(100);Pseudomonadales(100);Moraxellaceae(100);Moraxella(100);osloensis_oral_taxon_711(100); |
| 00182 | Ecotype_1 | 2.74325 | 0.0223199 | Bacteria(100);Bacteroidetes(100);Flavobacteria(100);Flavobacteriales(100);Flavobacteriaceae(99);Bergeyella(99);sp. |
| 00188 | Ecotype_1 | 2.61132 | 0.0110145 | Bacteria(100);Proteobacteria(100);Betaproteobacteria(100);Burkholderiales(100);Comamonadaceae(100);Hydrogenophaga(96);sp. |
| 00200 | Ecotype_1 | 2.57185 | 8.0888E-05 | Bacteria(100);Firmicutes(100);Clostridia(100);Clostridiales(100);unclassified(99);unclassified(99);sp. |
| 00219 | Ecotype_1 | 2.79268 | 0.0107125 | Bacteria(100);Actinobacteria(100);Actinobacteria(100);Actinomycetales(100);Micrococcaceae(100);Kocuria(100);sp._oral_taxon_189(100); |
| 00229 | Ecotype_1 | 2.62256 | 0.00033488 | Bacteria(100);Actinobacteria(100);Actinobacteria(100);Actinomycetales(100);Microbacteriaceae(94);Microbacterium(94);sp. |

**Supplementary Table 2**

**Differentially abundant OTUs between the sub-ecotypes A, B and C detected by a linear discriminant analysis (LDA) effect size (LEfSe) analysis**

This table shows all the OTUs detected as significantly differentially abundant and their taxonomic assignment and the corresponding bootstrap value (in brackets). No bootstrap values indicate that we did not find a confident match (cutoff 80) and therefore we stop the classification at the previous level.

|  | **Comparison BA** | | | **Comparison BC** | | | **Comparison AC** | | |  |
| --- | --- | --- | --- | --- | --- | --- | --- | --- | --- | --- |
| **Otu** | **Most abundant in** | **LDA** | **pValue** | **Most abundant in** | **LDA** | **pValue** | **Most abundant in** | **LDA** | **pValue** | **taxonomy** |
| 00001 | - |  |  | sub-ecotype A | 4.82644 | 4.8134E-06 | sub-ecotype B | 4.73425 | 5.8652E-06 | Bacteria(100);Fusobacteria(100);Fusobacteria(100);Fusobacteriales(100);Fusobacteriaceae(100);Fusobacterium(100);nucleatum_ss_vincentii_oral_taxon_200(100); |
| 00002 | sub-ecotype A | 4.16661 | 4.7637E-05 | sub-ecotype A | 4.24387 | 0.00018183 | - |  |  | Bacteria(100);Firmicutes(100);Bacilli(100);Lactobacillales(100);Streptococcaceae(100);Streptococcus(100);sp._oral_taxon_058(100); |
| 00003 | sub-ecotype A | 3.95836 | 0.0481524 | sub-ecotype A | 4.18872 | 0.00027495 | sub-ecotype B | 3.77259 | 0.00534392 | Bacteria(100);Proteobacteria(100);Gammaproteobacteria(100);Pasteurellales(100);Pasteurellaceae(100);Haemophilus(100);parainfluenzae_oral_taxon_718(84); |
| 00004 | sub-ecotype A | 4.08042 | 0.00023523 | sub-ecotype A | 4.14573 | 3.856E-05 | sub-ecotype B | 3.24604 | 0.0221726 | Bacteria(100);Proteobacteria(100);Betaproteobacteria(100);Neisseriales(100);Neisseriaceae(100);Neisseria(100);sp. |
| 00005 | - |  |  | sub-ecotype C | 4.7532 | 0.00019813 | sub-ecotype C | 4.75332 | 0.00055985 | Bacteria(100);Bacteroidetes(100);Bacteroides(100);Bacteroidales(100);Porphyromonadaceae(100);Porphyromonas(100);gingivalis_oral_taxon_619(100); |
| 00006 | - |  |  | sub-ecotype A | 3.67427 | 0.0395489 | - |  |  | Bacteria(100);Actinobacteria(100);Actinobacteria(100);Actinomycetales(100);Corynebacteriaceae(100);Corynebacterium(100);matruchotii_oral_taxon_666(96); |
| 00007 | sub-ecotype B | 3.94577 | 0.00823321 | - |  |  | - |  |  | Bacteria(100);Proteobacteria(100);Epsilonproteobacteria(100);Campylobacterales(100);Campylobacteraceae(100);Campylobacter(100);sp. |
| 00008 | sub-ecotype A | 3.64593 | 0.00086187 | - |  |  | - |  |  | Bacteria(100);Actinobacteria(100);Actinobacteria(100);Actinomycetales(100);Micrococcaceae(100);Rothia(100);dentocariosa_oral_taxon_587(94); |
| 00009 | sub-ecotype A | 3.68769 | 0.0325381 | sub-ecotype A | 3.56659 | 0.0305325 | - |  |  | Bacteria(100);Actinobacteria(100);Actinobacteria(100);Actinomycetales(100);Actinomycetaceae(100);Actinomyces(100);sp._oral_taxon_171(100); |
| 00012 | sub-ecotype B | 3.83966 | 0.00325824 | sub-ecotype C | 4.02717 | 4.605E-05 | - |  |  | Bacteria(100);Bacteroidetes(100);Bacteroides(100);Bacteroidales(100);Porphyromonadaceae(100);Tannerella(100);forsythia_oral_taxon_613(100); |
| 00016 | sub-ecotype B | 3.97004 | 0.00030695 | sub-ecotype C | 4.21024 | 2.9189E-05 | - |  |  | Bacteria(100);Spirochaetes(100);Spirochaetes(100);Spirochaetales(100);Spirochaetaceae(100);Treponema(100);denticola_oral_taxon_584(100); |
| 00017 | sub-ecotype B | 4.05348 | 0.00129639 | sub-ecotype C | 3.93386 | 0.00047885 | - |  |  | Bacteria(100);Spirochaetes(100);Spirochaetes(100);Spirochaetales(100);Spirochaetaceae(100);Treponema(100);sp. |
| 00018 | sub-ecotype A | 3.56949 | 0.0143251 | sub-ecotype A | 3.62172 | 0.00036496 | - |  |  | Bacteria(100);Proteobacteria(100);Betaproteobacteria(100);Burkholderiales(100);Burkholderiaceae(100);Lautropia(100);mirabilis_oral_taxon_022(100); |
| 00019 | sub-ecotype B | 4.12626 | 0.00542593 | - |  |  | sub-ecotype B | 3.97634 | 0.0118649 | Bacteria(100);Bacteroidetes(100);Bacteroides(100);Bacteroidales(100);Prevotellaceae(100);Alloprevotella(100);tannerae_oral_taxon_466(100); |
| 00020 | - |  |  | - |  |  | sub-ecotype B | 3.9966 | 0.00102053 | Bacteria(100);Bacteroidetes(100);Bacteroides(100);Bacteroidales(100);Prevotellaceae(100);Prevotella(100);nigrescens_oral_taxon_693(100); |
| 00021 | - |  |  | sub-ecotype A | 3.53048 | 0.00040645 | sub-ecotype B | 3.49766 | 0.00313208 | Bacteria(100);Fusobacteria(100);Fusobacteria(100);Fusobacteriales(100);Leptotrichiaceae(100);Leptotrichia(100);sp. |
| 00022 | sub-ecotype B | 4.18392 | 0.00405698 | - |  |  | sub-ecotype B | 4.1699 | 0.0125835 | Bacteria(100);Bacteroidetes(100);Bacteroides(100);Bacteroidales(100);Bacteroidales_[F-2](100);Bacteroidales_[G-2](100);sp._oral_taxon_274(100); |
| 00023 | sub-ecotype B | 3.65089 | 0.00757491 | sub-ecotype C | 4.08939 | 1.9714E-05 | sub-ecotype C | 3.90787 | 0.0177731 | Bacteria(100);Synergistetes(100);Synergistetes_[C-1](100);Synergistetes_[O-1](100);Synergistetes_[F-2](100);Fretibacterium(100);sp._oral_taxon_361(100); |
| 00024 | - |  |  | sub-ecotype A | 3.64856 | 0.00497226 | sub-ecotype B | 3.19154 | 0.047815 | Bacteria(100);Bacteroidetes(100);Flavobacteria(100);Flavobacteriales(100);Flavobacteriaceae(100);Capnocytophaga(100);sp. |
| 00025 | sub-ecotype A | 4.04876 | 0.00042793 | sub-ecotype A | 4.17021 | 0.00288944 | - |  |  | Bacteria(100);Firmicutes(100);Bacilli(100);Bacillales(100);Staphylococcaceae(100);Staphylococcus(100);warneri_oral_taxon_076(100); |
| 00026 | sub-ecotype A | 3.22331 | 0.00078942 | sub-ecotype A | 3.46829 | 1.6344E-05 | - |  |  | Bacteria(100);Firmicutes(100);Bacilli(100);Bacillales(100);Staphylococcaceae(100);Gemella(100);morbillorum_oral_taxon_046(100); |
| 00027 | sub-ecotype A | 3.29857 | 0.00469379 | - |  |  | - |  |  | Bacteria(100);Firmicutes(100);Clostridia(100);Clostridiales(100);Veillonellaceae(100);Veillonella(100);sp._oral_taxon_780(100); |
| 00028 | sub-ecotype B | 3.88283 | 0.00010201 | sub-ecotype C | 3.85939 | 0.00018183 | - |  |  | Bacteria(100);Spirochaetes(100);Spirochaetes(100);Spirochaetales(100);Spirochaetaceae(100);Treponema(100);maltophilum_oral_taxon_664(100); |
| 00029 | - |  |  | sub-ecotype A | 3.45725 | 0.00025477 | sub-ecotype B | 3.91908 | 0.00953862 | Bacteria(100);Proteobacteria(100);Gammaproteobacteria(100);Pasteurellales(100);Pasteurellaceae(100);Aggregatibacter(97);sp. |
| 00030 | sub-ecotype B | 3.83399 | 0.0202529 | - |  |  | - |  |  | Bacteria(100);Bacteroidetes(100);Bacteroides(100);Bacteroidales(100);Prevotellaceae(100);Prevotella(100);pleuritidis_[NV]_oral_taxon_303(99); |
| 00032 | - |  |  | sub-ecotype C | 3.89645 | 0.00459611 | - |  |  | Bacteria(100);Bacteroidetes(100);Bacteroidetes_[C-1](100);Bacteroidetes_[O-1](100);Bacteroidetes_[F-1](100);Bacteroidetes_[G-5](100);sp._oral_taxon_511(99); |
| 00033 | - |  |  | sub-ecotype A | 3.23405 | 0.00307226 | sub-ecotype B | 3.11968 | 0.0284518 | Bacteria(100);Fusobacteria(100);Fusobacteria(100);Fusobacteriales(100);Leptotrichiaceae(100);Leptotrichia(100);sp. |
| 00034 | - |  |  | sub-ecotype A | 3.53887 | 0.00051305 | - |  |  | Bacteria(100);Proteobacteria(100);Betaproteobacteria(100);Neisseriales(100);Neisseriaceae(100);Eikenella(99);corrodens_oral_taxon_577(99); |
| 00036 | sub-ecotype A | 3.5146 | 0.00059387 | sub-ecotype A | 3.53085 | 1.1786E-05 | - |  |  | Bacteria(100);Firmicutes(100);Bacilli(100);Lactobacillales(100);Enterococcaceae(100);Enterococcus(100);faecalis_oral_taxon_604(100); |
| 00037 | sub-ecotype A | 3.33266 | 0.00072138 | sub-ecotype A | 3.29219 | 6.307E-05 | - |  |  | Bacteria(100);Firmicutes(100);Bacilli(100);Bacillales(98);Bacillaceae(97);Anaerobacillus(94);sp. |
| 00038 | sub-ecotype B | 3.18571 | 0.0453423 | - |  |  | - |  |  | Bacteria(100);Firmicutes(100);Clostridia(100);Clostridiales(100);Veillonellaceae(100);Selenomonas(100);sp. |
| 00040 | - |  |  | sub-ecotype A | 3.22254 | 0.00039292 | sub-ecotype B | 2.85896 | 0.0108127 | Bacteria(100);Proteobacteria(100);Gammaproteobacteria(100);Cardiobacteriales(100);Cardiobacteriaceae(100);Cardiobacterium(100);hominis_oral_taxon_633(100); |
| 00041 | sub-ecotype A | 3.47919 | 0.00362506 | sub-ecotype A | 3.48878 | 0.00361874 | - |  |  | Bacteria(100);Proteobacteria(100);Gammaproteobacteria(100);Xanthomonadales(100);Xanthomonadaceae(100);Stenotrophomonas(88);maltophilia_oral_taxon_663(88); |
| 00042 | sub-ecotype A | 3.68106 | 0.00051222 | sub-ecotype A | 3.66987 | 4.0958E-05 | - |  |  | Bacteria(100);Firmicutes(100);Bacilli(100);Lactobacillales(100);Streptococcaceae(100);Streptococcus(100);sp._oral_taxon_058(100); |
| 00043 | sub-ecotype B | 3.50215 | 0.00047681 | sub-ecotype C | 3.47957 | 0.00010101 | - |  |  | Bacteria(100);Spirochaetes(100);Spirochaetes(100);Spirochaetales(100);Spirochaetaceae(100);Treponema(100);socranskii_oral_taxon_769(96); |
| 00044 | - |  |  | sub-ecotype C | 3.57087 | 0.00719706 | - |  |  | Bacteria(100);Spirochaetes(100);Spirochaetes(100);Spirochaetales(100);Spirochaetaceae(100);Treponema(100);sp._oral_taxon_257(80); |
| 00046 | - |  |  | sub-ecotype A | 2.73714 | 0.0127195 | - |  |  | Bacteria(100);Bacteroidetes(100);Flavobacteria(100);Flavobacteriales(100);Flavobacteriaceae(100);Capnocytophaga(100);granulosa_oral_taxon_325(100); |
| 00048 | sub-ecotype A | 2.98244 | 0.036101 | sub-ecotype A | 3.23426 | 0.00284257 | - |  |  | Bacteria(100);Firmicutes(100);Bacilli(100);Lactobacillales(100);Streptococcaceae(100);Streptococcus(100);vestibularis_oral_taxon_021(93); |
| 00049 | - |  |  | sub-ecotype A | 3.07049 | 0.0476906 | - |  |  | Bacteria(100);Bacteroidetes(100);Bacteroides(100);Bacteroidales(100);Porphyromonadaceae(100);Porphyromonas(100);sp._oral_taxon_279(98); |
| 00051 | sub-ecotype A | 2.7927 | 0.00822654 | sub-ecotype A | 2.7927 | 0.00725699 | - |  |  | Bacteria(100);Actinobacteria(100);Actinobacteria(100);Actinomycetales(100);Corynebacteriaceae(100);Corynebacterium(100);durum_oral_taxon_595(100); |
| 00052 | sub-ecotype B | 3.1954 | 0.0399788 | - |  |  | - |  |  | Bacteria(100);Firmicutes(100);Clostridia(100);Clostridiales(100);Veillonellaceae(100);Selenomonas(100);sputigena_oral_taxon_151(100); |
| 00053 | - |  |  | sub-ecotype C | 3.8455 | 0.00027236 | sub-ecotype C | 3.75903 | 0.00058273 | Bacteria(100);Bacteroidetes(100);Bacteroides(100);Bacteroidales(100);Prevotellaceae(100);Prevotella(100);sp._oral_taxon_526(100); |
| 00054 | sub-ecotype A | 3.22541 | 0.00266663 | sub-ecotype A | 3.19207 | 0.0464497 | sub-ecotype C | 2.66048 | 0.0195219 | Bacteria(100);Firmicutes(100);Bacilli(100);Bacillales(100);Bacillaceae(100);Bacillus(99);sp. |
| 00055 | sub-ecotype A | 2.93361 | 0.00248348 | sub-ecotype A | 3.00165 | 0.00059625 | - |  |  | Bacteria(100);Proteobacteria(100);Alphaproteobacteria(100);Rhodobacterales(100);Rhodobacteraceae(100);Paracoccus(89);sp. |
| 00056 | sub-ecotype B | 3.29726 | 0.00422943 | sub-ecotype C | 3.41623 | 0.0004678 | - |  |  | Bacteria(100);Firmicutes(100);Clostridia(100);Clostridiales(100);Peptostreptococcaceae_[XI](100);Filifactor(100);alocis_oral_taxon_539(100); |
| 00057 | sub-ecotype A | 2.82487 | 0.0385463 | sub-ecotype A | 2.93811 | 0.003542 | - |  |  | Bacteria(100);Bacteroidetes(100);Flavobacteria(100);Flavobacteriales(100);Flavobacteriaceae(100);Bergeyella(100);sp._oral_taxon_422(100); |
| 00059 | sub-ecotype B | 3.28271 | 0.0279123 | sub-ecotype C | 3.52132 | 0.0345406 | - |  |  | Bacteria(100);Tenericutes(100);Mollicutes(100);Mycoplasmatales(100);Mycoplasmataceae(100);Mycoplasma(100);faucium_oral_taxon_606(89); |
| 00060 | - |  |  | sub-ecotype A | 2.95113 | 0.0137941 | - |  |  | Bacteria(100);Verrucomicrobia(100);Opitutae(100);Opitutales(100);Opitutaceae(100);Opitutus(100);sp. |
| 00061 | sub-ecotype A | 2.52915 | 0.0472849 | sub-ecotype C | 3.60423 | 0.00749002 | sub-ecotype C | 3.68383 | 3.624E-05 | Bacteria(100);Proteobacteria(100);Deltaproteobacteria(100);Desulfovibrionales(100);Desulfomicrobiaceae(100);Desulfomicrobium(100);orale_oral_taxon_703(100); |
| 00062 | - |  |  | sub-ecotype A | 3.06942 | 0.00191648 | - |  |  | Bacteria(100);Firmicutes(100);Clostridia(100);Clostridiales(100);Peptostreptococcaceae_[XIII](100);Parvimonas(100);micra_oral_taxon_111(93); |
| 00063 | sub-ecotype B | 3.18797 | 0.0199453 | sub-ecotype C | 3.12279 | 0.00234666 | - |  |  | Bacteria(100);Synergistetes(100);Synergistetes_[C-1](100);Synergistetes_[O-1](100);Synergistetes_[F-2](100);Fretibacterium(100);fastidiosum_oral_taxon_363(100); |
| 00064 | sub-ecotype A | 2.95489 | 0.0154403 | sub-ecotype A | 3.04529 | 0.00374542 | - |  |  | Bacteria(100);Firmicutes(100);Bacilli(100);Lactobacillales(100);Carnobacteriaceae(100);Granulicatella(100);sp. |
| 00065 | sub-ecotype A | 3.2162 | 0.0275869 | sub-ecotype A | 3.25217 | 0.0465488 | - |  |  | Bacteria(100);Proteobacteria(100);Betaproteobacteria(100);Burkholderiales(100);Comamonadaceae(89);Comamonas(88);testosteroni_oral_taxon_858(88); |
| 00067 | - |  |  | sub-ecotype A | 3.3891 | 0.00218001 | sub-ecotype B | 2.69311 | 0.0201256 | Bacteria(100);Fusobacteria(100);Fusobacteria(100);Fusobacteriales(100);Leptotrichiaceae(100);Leptotrichia(100);goodfellowii_oral_taxon_845(99); |
| 00069 | - |  |  | sub-ecotype C | 3.52145 | 0.00689104 | sub-ecotype C | 3.45573 | 0.00646254 | Bacteria(100);Firmicutes(100);Clostridia(100);Clostridiales(100);Peptostreptococcaceae_[XIII](100);Peptostreptococcaceae_[XIII][G-1](100);sp._oral_taxon_113(100); |
| 00074 | - |  |  | sub-ecotype A | 2.82394 | 0.00039115 | - |  |  | Bacteria(100);Bacteroidetes(100);Flavobacteria(100);Flavobacteriales(100);Flavobacteriaceae(100);Bergeyella(100);sp._oral_taxon_322(100); |
| 00075 | - |  |  | sub-ecotype A | 2.73052 | 0.0149013 | - |  |  | Bacteria(100);Bacteroidetes(100);Flavobacteria(100);Flavobacteriales(100);Flavobacteriaceae(100);Capnocytophaga(100);sp._oral_taxon_326(95); |
| 00076 | sub-ecotype A | 3.00306 | 0.00937189 | sub-ecotype A | 3.00985 | 0.0146439 | - |  |  | Bacteria(100);Proteobacteria(100);Gammaproteobacteria(100);Pseudomonadales(100);Moraxellaceae(100);Acinetobacter(100);sp._oral_taxon_408(100); |
| 00078 | sub-ecotype A | 2.69905 | 0.00175668 | sub-ecotype A | 2.86301 | 2.5541E-05 | - |  |  | Bacteria(100);Actinobacteria(100);Actinobacteria(100);Micrococcales(100);Microbacteriaceae(75);unclassified(75); |
| 00080 | sub-ecotype A | 2.70388 | 0.0242893 | sub-ecotype A | 2.72051 | 0.00255113 | - |  |  | acteria(100);Firmicutes(100);Bacilli(100);Bacillales(100);Family_XII(100);Exiguobacterium(100);sp. |
| 00084 | sub-ecotype B | 2.77744 | 0.0221808 | - |  |  | - |  |  | Bacteria(100);Bacteroidetes(100);Bacteroides(100);Bacteroidales(100);Prevotellaceae(100);Alloprevotella(100);sp._oral_taxon_912(100); |
| 00086 | sub-ecotype B | 3.44215 | 0.00455864 | - |  |  | - |  |  | Bacteria(100);Bacteroidetes(100);Bacteroidia(100);Bacteroidales(100);Rikenellaceae(100);Blvii28_wastewater-sludge_group(100);sp. |
| 00088 | - |  |  | sub-ecotype A | 2.54946 | 0.0165774 | sub-ecotype B | 2.60981 | 0.0326484 | Bacteria(100);Bacteroidetes(100);Flavobacteria(100);Flavobacteriales(100);Flavobacteriaceae(100);Bergeyella(100);sp._oral_taxon_907(85); |
| 00089 | - |  |  | sub-ecotype A | 3.13419 | 0.00184996 | - |  |  | Bacteria(100);Firmicutes(100);Bacilli(100);Lactobacillales(100);Carnobacteriaceae(100);Granulicatella(100);elegans_oral_taxon_596(92); |
| 00090 | sub-ecotype B | 2.85919 | 0.0235901 | - |  |  | - |  |  | Bacteria(100);Firmicutes(100);Clostridia(100);Clostridiales(100);Peptostreptococcaceae(100);Incertae_Sedis(100);sp. |
| 00091 | sub-ecotype A | 2.67456 | 0.00225662 | sub-ecotype A | 2.82798 | 0.00019358 | - |  |  | Bacteria(100);Firmicutes(100);Bacilli(100);Lactobacillales(100);Aerococcaceae(100);Abiotrophia(100);defectiva_oral_taxon_389(100); |
| 00093 | - |  |  | sub-ecotype A | 3.00632 | 0.00999357 | sub-ecotype B | 3.06101 | 0.00104386 | Bacteria(100);Firmicutes(100);Clostridia(100);Clostridiales(100);Lachnospiraceae_[XIVa](100);Johnsonella(100);ignava_oral_taxon_635(100); |
| 00094 | - |  |  | sub-ecotype C | 2.96845 | 0.0283861 | - |  |  | Bacteria(100);Spirochaetes(98);Spirochaetes(98);Spirochaetales(98);Spirochaetaceae(98);Treponema(98);sp. |
| 00101 | - |  |  | sub-ecotype C | 3.30372 | 0.00297592 | sub-ecotype C | 3.23555 | 0.00327975 | Bacteria(100);Spirochaetes(100);Spirochaetes(100);Spirochaetales(100);Spirochaetaceae(100);Treponema(100);sp._oral_taxon_258(100); |
| 00102 | - |  |  | sub-ecotype A | 2.66274 | 0.0334898 | - |  |  | Bacteria(100);Fusobacteria(100);Fusobacteria(100);Fusobacteriales(100);Leptotrichiaceae(100);Leptotrichia(100);sp._oral_taxon_219(94); |
| 00103 | sub-ecotype B | 2.8168 | 0.0482211 | sub-ecotype C | 3.25901 | 0.00131635 | - |  |  | Bacteria(100);Firmicutes(100);Clostridia(100);Clostridiales(100);Lachnospiraceae_[XIVa](100);Lachnospiraceae_[G-8](100);sp._oral_taxon_500(100); |
| 00104 | sub-ecotype B | 3.01291 | 0.0101156 | sub-ecotype C | 2.8682 | 0.0357734 | - |  |  | Bacteria(100);Firmicutes(100);Clostridia(100);Clostridiales(100);Veillonellaceae(100);Veillonellaceae_[G-1](100);sp._oral_taxon_129(99); |
| 00113 | - |  |  | - |  |  | sub-ecotype B | 2.78781 | 0.0130402 | Bacteria(100);Firmicutes(100);Clostridia(100);Clostridiales(100);Peptostreptococcaceae_[XI](100);Eubacterium_[XI][G-3](100);brachy_oral_taxon_557(100); |
| 00116 | - |  |  | sub-ecotype C | 2.95209 | 0.0446258 | - |  |  | Bacteria(100);Firmicutes(100);Clostridia(100);Clostridiales(100);Clostridiales_[F-1](100);Clostridiales_[F-1][G-1](100);sp._oral_taxon_093(100); |
| 00117 | - |  |  | sub-ecotype C | 2.82138 | 0.00270021 | - |  |  | Bacteria(100);Proteobacteria(100);Deltaproteobacteria(100);Desulfobacterales(100);Desulfobulbaceae(100);Desulfobulbus(100);sp._oral_taxon_041(100); |
| 00124 | sub-ecotype B | 3.05391 | 0.0294419 | - |  |  | - |  |  | Bacteria(100);Spirochaetes(100);Spirochaetes(100);Spirochaetales(100);Spirochaetaceae(100);Treponema(100);sp._oral_taxon_239(91); |
| 00125 | sub-ecotype A | 2.77022 | 0.00157773 | sub-ecotype A | 2.84759 | 0.00077864 | - |  |  | Bacteria(100);Proteobacteria(100);Betaproteobacteria(100);Burkholderiales(100);Comomonadaceae(100);Delftia(100);acidovorans_oral_taxon_023(100); |
| 00128 | sub-ecotype B | 2.80839 | 0.00223403 | sub-ecotype C | 2.79238 | 0.0167889 | - |  |  | Bacteria(100);Firmicutes(100);Clostridia(100);Clostridiales(100);Veillonellaceae(100);Veillonellaceae_[G-1](100);sp._oral_taxon_155(100); |
| 00130 | sub-ecotype A | 2.56415 | 0.0422564 | sub-ecotype A | 2.63304 | 0.00744233 | - |  |  | Bacteria(100);Proteobacteria(100);Gammaproteobacteria(100);Pseudomonadales(100);Pseudomonadaceae(100);Pseudomonas(100);sp._oral_taxon_032(81); |
| 00132 | - |  |  | sub-ecotype A | 2.45328 | 0.0448825 | - |  |  | Bacteria(100);Proteobacteria(100);Gammaproteobacteria(100);Oceanospirillales(100);Halomonadaceae(100);Halomonas(100);sp. |
| 00137 | - |  |  | sub-ecotype A | 2.66539 | 0.0310092 | - |  |  | Bacteria(100);Firmicutes(100);Clostridia(100);Clostridiales(100);Peptostreptococcaceae_[XI](100);Peptostreptococcus(100);stomatis_oral_taxon_112(100); |
| 00139 | sub-ecotype B | 2.43461 | 0.0257482 | sub-ecotype C | 3.03711 | 0.00752345 | - |  |  | Bacteria(100);Bacteroidetes(100);Bacteroides(100);Bacteroidales(100);Bacteroidaceae(100);Bacteroidaceae_[G-1]_(100);sp._oral_taxon_272(100); |
| 00140 | - |  |  | sub-ecotype C | 2.62573 | 0.0395741 | - |  |  | Bacteria(100);SR1(100);SR1_[C-1](100);SR1_[O-1](100);SR1_[F-1](100);SR1_[G-1](100);sp._oral_taxon_874(99); |
| 00141 | - |  |  | sub-ecotype C | 2.65613 | 0.0429151 | - |  |  | Bacteria(100);Firmicutes(100);Clostridia(100);Clostridiales(100);Peptostreptococcaceae_[XI](100);Peptostreptococcaceae_[XI][G-4](100);sp. |
| 00142 | sub-ecotype A | 2.76955 | 0.0307973 | - |  |  | - |  |  | Bacteria(100);Firmicutes(100);Clostridia(100);Clostridiales(100);Veillonellaceae(100);Veillonella(100);sp._oral_taxon_780(100); |
| 00147 | - |  |  | sub-ecotype A | 2.72851 | 0.00643739 | sub-ecotype B | 2.70777 | 0.0108466 | Bacteria(100);Bacteroidetes(100);Flavobacteria(100);Flavobacteriales(100);Flavobacteriaceae(100);Capnocytophaga(100);sp._oral_taxon_338(99); |
| 00148 | - |  |  | sub-ecotype A | 2.87782 | 0.00170406 | - |  |  | Bacteria(100);Bacteroidetes(100);Bacteroidia(100);Bacteroidales(100);Porphyromonadaceae(100);Paludibacter(100);sp. |
| 00156 | - |  |  | sub-ecotype C | 2.84533 | 0.00910401 | - |  |  | Bacteria(100);Bacteroidetes(100);Bacteroidetes_[C-1](100);Bacteroidetes_[O-1](100);Bacteroidetes_[F-1](100);Bacteroidetes_[G-3](100);sp._oral_taxon_280(100); |
| 00161 | sub-ecotype A | 2.55381 | 0.00147796 | sub-ecotype A | 2.61407 | 0.00023998 | - |  |  | Bacteria(100);Proteobacteria(100);Gammaproteobacteria(100);Pseudomonadales(100);Pseudomonadaceae(100);Pseudomonas(100);sp._oral_taxon_032(81); |
| 00163 | sub-ecotype A | 2.50499 | 0.00135008 | sub-ecotype A | 2.66212 | 0.00308164 | - |  |  | Bacteria(100);Proteobacteria(100);Betaproteobacteria(100);Burkholderiales(100);Oxalobacteraceae(100);Janthinobacterium(61);sp. |
| 00169 | - |  |  | sub-ecotype C | 2.82618 | 0.0395999 | - |  |  | Bacteria(100);Spirochaetes(100);Spirochaetes(100);Spirochaetales(100);Spirochaetaceae(100);Treponema(100);sp._oral_taxon_270(100); |
| 00173 | sub-ecotype A | 2.54504 | 0.00058463 | sub-ecotype A | 2.58328 | 0.000258 | - |  |  | Bacteria(100);Candidate_division_OD1(100);unclassified(100) |
| 00175 | sub-ecotype A | 2.48982 | 0.01091 | sub-ecotype A | 2.61804 | 0.00021696 | - |  |  | Bacteria(100);Proteobacteria(100);Gammaproteobacteria(100);Pseudomonadales(100);Moraxellaceae(100);Moraxella(100);osloensis_oral_taxon_711(100); |
| 00180 | - |  |  | sub-ecotype C | 2.76722 | 0.0143423 | sub-ecotype C | 2.81046 | 0.00751526 | Bacteria(100);Proteobacteria(100);Deltaproteobacteria(100);Desulfovibrionales(100);Desulfovibrionaceae(100);Desulfovibrio(100);fairfieldensis_oral_taxon_605(85); |
| 00182 | - |  |  | sub-ecotype A | 2.44018 | 0.00084299 | - |  |  | Bacteria(100);Bacteroidetes(100);Flavobacteria(100);Flavobacteriales(100);Flavobacteriaceae(99);Bergeyella(99);sp. |
| 00186 | sub-ecotype B | 2.45758 | 0.0167034 | - |  |  | - |  |  | Bacteria(100);Firmicutes(100);Clostridia(100);Clostridiales(100);Veillonellaceae(100);Anaeroglobus(99);geminatus_oral_taxon_121(99); |
| 00193 | sub-ecotype A | 2.56339 | 0.00061978 | sub-ecotype A | 2.55617 | 6.7376E-05 | - |  |  | Bacteria(100);Proteobacteria(100);Alphaproteobacteria(100);Sphingomonadales(100);Sphingomonadaceae(100);Sphingomonas(100);sp._oral_taxon_003(100); |
| 00196 | sub-ecotype A | 2.61411 | 0.0118334 | - |  |  | - |  |  | Bacteria(100);Proteobacteria(100);Gammaproteobacteria(100);Pseudomonadales(100);Pseudomonadaceae(100);Pseudomonas(100);sp. |
| 00217 | sub-ecotype B | 2.48734 | 0.0130515 | - |  |  | - |  |  | Bacteria(100);Firmicutes(100);Clostridia(100);Clostridiales(100);Veillonellaceae(100);Megasphaera(100);micronuciformis_oral_taxon_122(100); |
| 00222 | - |  |  | sub-ecotype C | 2.6336 | 2.7199E-05 | sub-ecotype C | 2.80441 | 0.0002019 | Bacteria(100);Bacteroidetes(100);Bacteroides(100);Bacteroidales(100);Porphyromonadaceae(100);Porphyromonas(100);gingivalis_oral_taxon_619(100); |
| 00223 | sub-ecotype B | 2.45679 | 0.0012115 | sub-ecotype C | 2.55997 | 2.713E-05 | - |  |  | Bacteria(100);Spirochaetes(100);Spirochaetes(100);Spirochaetales(100);Spirochaetaceae(100);Treponema(100);parvum_oral_taxon_724(100); |
| 00224 | - |  |  | - |  |  | sub-ecotype B | 2.4939 | 0.0159033 | Bacteria(100);Bacteroidetes(100);Bacteroides(100);Bacteroidales(100);Prevotellaceae(100);Prevotella(100);sp._oral_taxon_301(100); |
| 00226 | - |  |  | sub-ecotype C | 2.57001 | 0.00751526 | sub-ecotype C | 2.6672 | 0.0129052 | Bacteria(100);Firmicutes(100);Clostridia(100);Clostridiales(100);Lachnospiraceae_[XIVa](100);Moryella(100);sp._oral_taxon_373(100); |
| 00242 | - |  |  | - |  |  | sub-ecotype B | 2.55344 | 0.031973 | Bacteria(100);Bacteroidetes(100);Bacteroides(100);Bacteroidales(100);Prevotellaceae(100);Prevotella(100);marshii_oral_taxon_665(100); |
| 00250 | sub-ecotype B | 2.48693 | 0.00140876 | - |  |  | sub-ecotype B | 2.61153 | 0.00739296 | Bacteria(100);Firmicutes(100);Erysipelotrichi(100);Erysipelotrichales(100);Erysipelotrichaceae(100);Bulleidia(100);extructa_oral_taxon_603(100); |
| 00294 | sub-ecotype B | 2.37788 | 0.0118334 | - |  |  | - |  |  | Bacteria(100);Firmicutes(100);Erysipelotrichi(100);Erysipelotrichales(100);Erysipelotrichaceae(100);Erysipelotrichaceae_[G-1](100);sp._oral_taxon_904(96); |
